# Therapeutic Potential of *Origanum majorana* L. Essential Oil in Diabetes Mellitus: Insights From GC–MS Characterization, In Vivo Hypoglycaemic Studies, and In Silico Analyses

**DOI:** 10.1002/cbdv.71277

**Published:** 2026-05-04

**Authors:** Mahmoud Houas, Mohammed Larbi Benamor, Elhafnaoui Lanez, Yahia Bekkar, Lotfi Bourougaa, Ouafa Boudebia, Rania Bouraoui, Aicha Adaika, Nafila Zouaghi, Salah Neghmouche Nacer, Lazhar Bechki, Touhami Lanez, Stefania Garzoli

**Affiliations:** ^1^ Laboratory For the Study and Development of Water Treatment and Purification Techniques and Environmental Management Higher Normal School of Kouba Kouba Algeria; ^2^ Faculty of Exact Sciences Department of Chemistry VTRS Laboratory University of El Oued El Oued Algeria; ^3^ Faculty of Exact and Natural Sciences Department of Matter Sciences LMCE Laboratory University of Biskra Biskra Algeria; ^4^ Faculty of Material Sciences Department of Chemistry LCCE Laboratory University of Batna‐1 Batna Algeria; ^5^ Department of Chemistry Faculty of Exact Sciences University of El Oued El Oued Algeria; ^6^ VPRS Laboratory Department of Chemistry Faculty of Mathematics and Material Sciences Kasdi Merbah University Ouargla Algeria; ^7^ Department of Chemistry and Technologies of Drug Sapienza University Rome Italy

**Keywords:** ADMET analysis, DFT analysis, GC‐MS analysis, MM‐PBSA, molecular docking, molecular dynamics simulations

## Abstract

Diabetes mellitus is a widespread metabolic disorder characterized by impaired glucose regulation. This study investigated the chemical composition and antidiabetic potential of *Origanum majorana* essential oil (EO) using integrated in vitro, in vivo, and computational approaches. GC–MS analysis identified 42 constituents representing 96.28% of the oil, with trans‐thujone (33.30%), santolina triene (16.42%), and cis‐verbenyl acetate (15.05%) as the dominant components. In vitro assays revealed strong inhibitory activity against carbohydrate‐hydrolyzing enzymes, with IC_50_ values of 3.68 µg/mL for α‐amylase and 4.71 µg/mL for α‐glucosidase, which were lower than those of the reference drug acarbose (11.17 and 9.68 µg/mL, respectively). In vivo evaluation in alloxan‐induced diabetic rats demonstrated a significant reduction in fasting blood glucose levels from 1.47 ± 0.04 g/L in the diabetic group to 0.94 ± 0.03 g/L after EO treatment, accompanied by improvements in biochemical and histopathological parameters. Molecular docking identified several major EO constituents with strong binding affinities toward α‐amylase and α‐glucosidase, particularly cis‐verbenyl acetate (–6.64 and –7.27 kcal/mol) and β‐pinene oxide (–5.40 and –6.46 kcal/mol), exceeding the affinity of acarbose. ADMET analysis predicted favorable pharmacokinetic profiles and low toxicity risks for these compounds. Molecular dynamics simulations confirmed stable protein–ligand interactions, while MM‐PBSA calculations supported strong binding free energies. Density functional theory (DFT) analysis further revealed moderate reactivity and enhanced stability in aqueous environments. Overall, the combined experimental and computational findings suggest that *O. majorana* EO, particularly its constituents cis‐verbenyl acetate and β‐pinene oxide, represents a promising natural source of antidiabetic agents warranting further pharmacological investigation.

## Graphical abstract

Exploring the chemical profile and biological property of *O. majorana* EO.

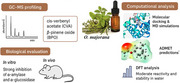

## 1 Introduction

Type 2 diabetes mellitus (T2DM) is a widespread endocrine disorder produced by deficient or defective insulin function. The disorder is often preceded by quantitative or qualitative failure of pancreatic β‐cells with specific relevance to hepatic, adipose, and skeletal muscle tissue and is hence contributing to reduced insulin sensitivity, endocrine dysregulation, and persistent hyperglycemia [1, 2]. Among the most documented pharmacologic strategies for treating postprandial hyperglycemia in patients with T2DM is inhibiting the digestion of dietary carbohydrate [3]. In the human GI tract, α‐amylase catalyzes the hydrolysis of polysaccharides to oligosaccharides and disaccharides, which are hydrolyzed into glucose by α‐glucosidase for absorption into the bloodstream [4]. Inhibition of these enzymes is thus one possible method for slowing carbohydrate digestion and controlling glycemic response [5].

Several synthetic pharmacologic agents such as metformin, sulfonylureas, thiazolidinediones, biguanides, meglitinides, and dipeptidyl peptidase‐IV inhibitors are used in the clinical management of T2DM [6, 7]. Chronic use of these agents is also usually accompanied by side effects in the nature of hepatotoxicity, hypoglycemia, and gastrointestinal distress [8]. Therefore, there is an increasing necessity to find and develop other therapeutic drugs that are effective as well as possess a good safety profile. In this context, medicinal plants are being investigated with immense interest due to their bioactive principles, pharmacological specificity, general availability, and relatively lower risk of toxicity [8, 9].

Of the plant‐based bioactive compounds, essential oils (EOs)—volatile, intensely aromatic, very concentrated plant compounds—have displayed a wide spectrum of therapeutic actions. Traditionally used in aromatherapy, cosmetology, and conventional medicine, EOs are presently being considered for their antidiabetic action primarily because of the intrinsic phytochemicals which can modulate glucose homeostasis, enhance insulin sensitivity, and mitigate oxidative stress [10].

*Origanum majorana* L. (Lamiaceae), commonly known as sweet marjoram, is an aromatic medicinal plant widely distributed in Mediterranean regions. Its essential oil is rich in monoterpenes and oxygenated monoterpenes such as thujones, terpinene derivatives, camphor, and eucalyptol, which are associated with several pharmacological properties including antioxidant, antimicrobial, anti‐inflammatory, and digestive effects. These biological activities have attracted increasing interest in the potential therapeutic applications of this species.

This study seeks is the determination of the antidiabetic potential of *O. majorana* L., which is a local medicinal plant of Algerian origin found in Tebessa province in Algeria. Hydrodistillation in a Clevenger‐type apparatus was used to obtain the essential oil, while the phytochemical components were determined by gas chromatography–mass spectrometry (GC‐MS). Biological activity of the EO was assayed using in vitro and in vivo assays and, in particular, its inhibitory activity on α‐amylase and α‐glucosidase was investigated. Besides, the study used computational methods as an adjunct and a complement to experimental findings.

## 2 Materials and Methods

### 2.1 Plant Material

The aerial parts of *O. majorana* L. were collected from the Negrine region of Tébessa Province, southeastern Algeria, between March and April 2023. The plant species was taxonomically identified by Prof. Atef CHOUIKH, Department of Biology, University of El Oued, Algeria. A voucher specimen of the plant material was deposited in the Herbarium of the Faculty of Natural and Life Sciences, University of El Oued, under the accession number [voucher no.: OMA‐2023‐79] for future reference.

The collection site is characterized by an arid desert climate at an elevation of approximately 58 m above sea level, located at 33°N latitude and 6°7′E longitude.

### 2.2 Essential Oil Extraction

The extraction of (EO) was carried out using a Clevenger‐type apparatus to ensure optimal yield and purity. A precisely weighed 100 g sample of plant material was thoroughly washed with distilled water before being placed in a heating flask with boiling regulators. A plant‐to‐water ratio of 1:10 (w/v) was used during the hydrodistillation process. Steam distillation was performed at 100°C for 3.5 h.

After distillation, the EO was separated from the aqueous phase through liquid‐liquid extraction using diethyl ether. The organic phase was then dried over anhydrous sodium sulfate and filtered to remove any residual solvent. To eliminate traces of diethyl ether, the oil was further concentrated using rotary evaporation. The final EO was stored in amber glass bottles at 5°C to maintain its chemical stability and integrity.

### 2.3 Gas Chromatography‐Mass Spectrometry Analysis

The chemical composition of the EO was analyzed using GC‐MS on an HP 5890 Series II GC system coupled to an HP‐MSD 5972 mass spectrometer. A 1 µL aliquot of the EO was injected in split mode (1:50), with helium (He) as the carrier gas at a constant flow rate of 1 mL/min. Separation was performed using an HP‐5 MS capillary column (30 m × 0.25 mm × 0.25 µm). Mass spectra were recorded at an ionization energy of 70 eV.

The temperature program for the column oven began at 60°C, where it was held for 8 min, followed by a gradual increase to 250°C at a rate of 2°C/min, maintained for 15 min. A final ramp increased the temperature to 300°C at 10°C/min.

The identification of EO components was achieved by comparing their mass spectra with the NIST 20 mass spectral database and Wiley libraries [11].

### 2.4 In Vivo Evaluation of the Antidiabetic Activities

#### 2.4.1 Animals Care

Twenty‐five adult male albino Wistar rats (150–200 g) were obtained from the Pasteur Institute (Algiers, Algeria). Upon arrival, animals were acclimatized for one week under controlled laboratory conditions (temperature 25°C ± 2°C; relative humidity 50%–60%; 12‐h light/dark cycle) with free access to standard pellet diet and water ad libitum.

Following acclimatization, animals were randomly allocated into five experimental groups (*n* = 6 per group) using a random number–based allocation method to minimize selection bias. Each group was housed in separate polypropylene cages containing sterile sawdust bedding under identical environmental conditions.

The selected sample size (*n* = 5 per group) was based on commonly accepted standards in alloxan‐induced antidiabetic in vivo studies and was considered sufficient to detect statistically significant differences in fasting blood glucose and related biochemical parameters at a significance level of *α* = 0.05, while adhering to the ethical principle of reduction in animal experimentation.

All experimental procedures involving animals were conducted in accordance with internationally accepted guidelines for the care and use of laboratory animals. The experimental protocol was reviewed and approved by the Institutional Animal Ethics Committee (IAEC) of the Faculty of Natural and Life Sciences, University of El Oued, El Oued, Algeria (approval no.: IAEC‐UEL‐2023‐008). All procedures complied with national regulations governing animal experimentation and were designed to minimize animal suffering.

#### 2.4.2 Induction of Hyperglycemia

Hyperglycemia was induced in experimental rats by a single intraperitoneal (IP) injection of freshly prepared alloxan monohydrate at a dose of 150 mg/kg body weight following an overnight fasting period to enhance diabetogenic efficiency. Alloxan selectively destroys pancreatic β‐cells through the generation of reactive oxygen species, leading to persistent hyperglycemia. However, its administration may initially induce a transient hypoglycemic phase due to the sudden release of insulin from damaged β‐cells. To prevent severe hypoglycemic shock during this early phase, a small supportive dose of insulin was administered to stabilize blood glucose levels and reduce mortality among the experimental animals.

In addition, the rats were provided with glucose‐enriched drinking water consisting of a 20 % glucose solution during the first 24 h, followed by a 5 % glucose solution for the subsequent 2 days. 72 h after alloxan administration, blood glucose levels were measured using a glucometer. Rats with blood glucose levels exceeding 14 mmol/L were considered diabetic and selected for further experimentation.

#### 2.4.3 Experimental Design

Following the confirmation of induction of diabetes, the animals were randomly distributed into five groups: Group 1 (control) consisted of healthy rats that were maintained on a normal diet. Group 2 (diabetic control) comprised diabetic rats that received only the standard diet but no medication. Group 3 (diabetic + acarbose) included diabetic rats that received acarbose along with their normal diet as a control for antidiabetic activity. Group 4 (diabetic + EO) comprised diabetic rats received *O. majorana* EO at a dose of approximately 100 mg/kg body weight (equivalent to 10 µL EO based on an estimated density of 0.9 g/mL). In addition, Group 5 (EO) consisted of non‐diabetic rats treated with *O. majorana* EO without diabetes induction. The treatment period started 72 h after diabetes induction and continued for 7 consecutive days to evaluate the impact of the *O. majorana* EO on glucose regulation.

#### 2.4.4 Euthanasia Procedure and Tissue Collection

The animals were fasted for 16 h at the end of treatment prior to anesthetization under 94% inhalation of chloroform. Decapitation was employed as a method of euthanasia, and blood glucose was assessed at sacrifice. The blood was taken in EDTA tubes for hematological examination, while other samples were placed in anticoagulant‐free tubes for application in biochemistry. The non‐anticoagulated tubes were spun at 3000 rpm for 10 min to provide serum, which was placed in the refrigerator at −4°C for further analysis of glycemia, urea, creatinine, triglycerides, aspartate aminotransferase (AST), alanine aminotransferase (ALT), and alkaline phosphatase (ALP) levels. The pancreas was dissected with caution after euthanasia, being careful to excise fat tissue surrounding it. The organ was then rinsed with a 0.9% NaCl solution to clear any remaining blood traces. Pancreatic tissue samples of each experimental group were then fixed in formalin solution to prepare for histological examination and preserve the best possible tissue integrity for future microscopic viewing.

### 2.5 In Vitro Evaluation of the Antidiabetic Activities

#### 2.5.1 α‐Amylase Inhibition Assay

α‐Amylase inhibition assay was conducted based on the protocol proposed by Deveci et al. [12], with slight modifications to optimize the procedure. A reaction mixture was established based on 25 µL of phosphate buffer (20 mM, pH 6.9) with α‐amylase (0.5 mg/mL) blended with an equivalent amount of EO samples or acarbose in varied concentrations (1, 25, 50, 100, 200, and 400 µg/mL). The mixture was pre‐incubated at 25°C for 10 min to allow the interaction between the inhibitors and the enzyme. Subsequent to pre‐incubation, the reaction was initiated by adding 25 µL of a 0.5% starch solution dissolved in the same phosphate buffer. The reaction was allowed to continue at 25°C for 30 min and then 50 µL of 3,5‐dinitrosalicylic acid (96 mM) was added to inhibit the enzymatic activity. The reaction tubes were placed in a water bath for 5 min and then cooled to room temperature. The absorbance of all the samples was measured at 540 nm in a spectrophotometer to determine the level of inhibition of α‐amylase.

#### 2.5.2 α‐Glucosidase Inhibition Assay

The α‐glucosidase inhibition assay was performed as per the protocol of Kim et al. [13] with slight modifications for improved accuracy. A reaction mixture was prepared by adding 50 µL of EO samples or acarbose at various concentrations (1, 25, 50, 100, 200, and 400 µg/mL) to 100 µL of Tris buffer (20 mM, pH 6.8). 100 µL of crude α‐glucosidase enzyme solution (0.01 mg/mL) was added to this mixture and the solution pre‐incubated at 25°C for 10 min to facilitate enzyme‐inhibitor interactions.

Next, the enzymatic reaction was triggered by the addition of 50 µL of 5 mM p‐nitrophenyl‐α‐D‐glucopyranoside (PNPg) and incubation at 37°C for 15 min. The reaction was halted by adding 2 mL of 500 mM sodium carbonate (Na_2_CO_3_), which gives yellow color upon formation. The resulting solution's absorbance was read at 405 nm using a spectrophotometer to quantify the inhibitory activity against α‐glucosidase.

Percentage inhibition (I %) of α‐amylase as well as α‐glucosidase was calculated by using the following formula:

(1) $$I\;\%=\frac{A_{0}-A}{A_{0}}\;\times \;100$$

where, A_0_ represents the absorbance of the control and A represents the absorbance of the sample.

A control reaction, representing 100% enzymatic activity, was conducted using distilled water instead of the sample.

### 2.6 In Silico Evaluation of the Antidiabetic Activity

#### 2.6.1 Molecular Docking Studies

Molecular docking simulations were carried out to investigate the binding interactions of the predominant constituents (abundance >1%) identified in *O. majorana* EO with two key enzymes regulating carbohydrate metabolism: α‐amylase (PDB ID: 3BAJ) and α‐glucosidase (PDB ID: 3A4A). The docking studies were conducted using AutoDock Tools (ADT) version 1.5.7 [14].

Protein structures were retrieved from the Protein Data Bank (PDB) [15] and prepared by eliminating water molecules and co‐crystallized residues. The ligand structures were obtained from PubChem in SDF format and converted to PDB format via Open Babel [16, 17]. Receptor preparation in ADT involved the addition of polar hydrogen atoms and Kollman charges [18, 19, 20, 21].

The ligands were assigned flexible torsions, while a defined grid box was set around the enzyme active site for docking: 50 × 40 × 40 Å for α‐amylase and 40 × 40 × 40 Å for α‐glucosidase. The grid centers were positioned at X = 8.652, Y = 15.386, Z = 40.189 for α‐amylase and X = 21.544, Y = ‐7.476, Z = 24.158 for α‐glucosidase.

Docking simulations were performed using the Lamarckian Genetic Algorithm with 100 independent runs per ligand, keeping all other parameters at default settings. Post‐docking analysis and visualization were conducted using Discovery Studio 2021 [22]. To ensure the reliability of the docking results, a redocking procedure was performed by reintroducing the native co‐crystallized ligand into the enzyme's binding pocket. The docking protocol was deemed valid when the root mean square deviation (RMSD) was below 2 Å [23].

#### 2.6.2 Molecular Dynamics (MD) Simulation

MD simulations of the protein‐ligand complexes with α‐amylase and α‐glucosidase were conducted using the GROMACS 2023 package over a 100 ns timeframe. The ligand topology files were generated using the SwissParam server, obtaining appropriate structural and force field parameters for the compounds [24]. The protein topologies were treated with the CHARMM36 all‐atom force field, which provided an accurate and detailed description of the enzyme structures [25, 26]. Each complex was solvated in a cubic box using the TIP3P water model, and Na+ and Cl‐ ions were added in order to neutralize the system. This was done to simulate the physiological condition for accurate biomolecular simulations [27]. Energy minimization was performed using the steepest descent algorithm until the maximal force was reduced below 10.0 kJ/mol, in order to achieve stability in the system before the simulation phase [20, 28].

The equilibration process was carried out in two steps for the system's stability before production MD simulations. Initially, NVT equilibration was first performed at 300 K for 100 ps using the v‐rescale thermostat with a coupling coefficient of 0.1 ps. This step allowed an opportunity for the system to reach thermal equilibrium while maintaining a constant volume. Subsequently, NPT equilibration was conducted for another 100 ps using the Berendsen barostat with a coupling constant of 2.0 ps, ensuring pressure stabilization before transitioning to the production phase [29, 30, 31].

Following equilibration, production MD simulations were conducted to analyze the structural stability and dynamic behavior of the complexes. The Root Mean Square Deviation (RMSD) was computed to examine the overall system stability throughout the simulation, while the Root Mean Square Fluctuation (RMSF) was utilized to obtain information about residue‐specific flexibility. The Radius of Gyration (Rg) was examined to determine the compactness of the complex, and the Solvent Accessible Surface Area (SASA) was calculated to examine exposure level of the protein to the solvent. These parameters collectively provided a comprehensive understanding of the conformational stability and molecular interactions within the system.

Molecular mechanics–Poisson–Boltzmann surface area (MM‐PBSA) analysis was employed to evaluate the binding interactions between the most active natural inhibitors and α‐amylase or α‐glucosidase. This method provides insights into binding energies, electrostatic interactions, solvation effects, and the overall stability of the complexes, offering valuable information on the molecular forces governing inhibitor efficacy [32]. The g_mmpbsa tool was used to calculate the binding energy of the receptor‐ligand complex, incorporating contributions from van der Waals interactions, electrostatic energies, solvation effects, and other critical energetic terms. This comprehensive approach enabled a detailed assessment of binding affinity and stability, elucidating the molecular basis of receptor‐ligand interactions [33]. The binding free energy (ΔG_binding) was determined using the equation:

(2) $$\Delta G_{Binding}=G_{complex}-\left(G_{protein}+G_{ligand}\right)$$

where *G_complex_* represents the total free energy of the protein‐ligand complex, and G_protein_ and G_ligand_ correspond to the total free energies of the enzyme and the inhibitor, respectively.

#### 2.6.3 ADMET Studies

ADMET (Absorption, Distribution, Metabolism, Excretion, and Toxicity) predictions were conducted to assess the pharmacokinetic and toxicity profiles of the major bioactive compounds identified in *O. majorana* EO. The analysis aimed to evaluate drug‐likeness, bioavailability, and safety parameters, providing insights into their potential as therapeutic agents. The SwissADME platform [34] was used to predict physicochemical properties, gastrointestinal absorption, blood‐brain barrier permeability, and cytochrome P450 interactions, while ProTox‐II [35] was employed to estimate toxicity risks, including hepatotoxicity, mutagenicity, and carcinogenicity.

#### 2.6.4 DFT Calculations

To explore the electronic properties of the key compounds in *O. majorana* EO, DFT calculations were performed using Gaussian 16 W software [36]. The molecular structures were optimized at the B3LYP/6‐311++G(d,p) level of theory [37], followed by frequency calculations to confirm the absence of imaginary frequencies, ensuring that all structures correspond to true energy minima.

To assess the reactivity and stability of the compounds, Frontier Molecular Orbital (FMO) analysis was conducted, providing insights into the HOMO‐LUMO energy levels and their corresponding energy gaps (ΔE). Additionally, Molecular Electrostatic Potential (MEP) mapping was used to visualize charge distribution, highlighting key regions for electrophilic and nucleophilic interactions.

The influence of solvation was evaluated using the Self‐Consistent Reaction Field (SCRF) method within the conductor‐like polarizable continuum model (CPCM) in water [38]. The obtained electronic parameters were further analyzed to understand the role of solvation in modulating molecular interactions.

All molecular structures, electronic charge distributions, and MEP surfaces were analyzed using GaussView 06 software [39], offering a detailed visualization of the compounds’ reactivity and interaction potential.

### 2.7 Statistical Analysis

All experimental data were expressed as mean ± standard deviation (SD). Statistical analyses were performed to evaluate differences between experimental groups. Prior to statistical testing, the datasets were examined for potential outliers and normal distribution. Comparisons among groups were conducted using one‐way analysis of variance (ANOVA) followed by Tukey's post hoc test to determine statistically significant differences between groups. The level of statistical significance was set at *p* < 0.05. All statistical analyses were performed using GraphPad Prism software (version 10.0, GraphPad Software Inc., USA) [40].

## 3 Results and Discussion

### 3.1 Chemical Composition of *O. majorana* EO

The EO of *O. majorana* was extracted from its aerial parts using hydrodistillation, yielding 1.45% (v/w).

GC‐MS analysis, identified a total of 42 major components, accounting for 96.28% of the total EO composition (Table 1). The major constituents identified were trans‐thujone (33.30%), santolina triene (16.42%), cis‐verbenyl acetate (15.05%), eucalyptol (10.71%), β‐pinene oxide (4.42%), sabinene (3.12%), camphor (2.85%), α‐thujene (1.44%), and α‐pinene (1.19%).

**TABLE 1:** Chemical composition of *O. majorana* EO.

No.	Compound	Area (%)	RI (Exp)	RI (Lit)	No.	Compound	Area (%)	RI (Exp)	RI (Lit)
1	Pentanol	0.12	759	762	22	Terpinene (unspecified)	0.76	1059	—
2	cis‐2‐Penten‐1‐ol	0.03	762	764	23	Sabinene hydrate	0.89	1068	1065
3	Hexanal	0.02	801	802	24	*p*‐Mentha‐2,4‐diene	0.11	1088	—
4	(Z)‐Salvene	0.02	844	—	25	Trans‐sabinene hydrate	0.86	1100	1098
5	Isopentyl acetate	0.02	869	871	26	Isopentyl 2‐methylbutanoate	0.14	1104	—
6	Santolina triene	16.42	898	—	27	β‐Thujone	0.24	1107	1110
7	Tricyclene	0.02	912	912	28	trans‐Thujone	33.30	1119	1120
8	α‐Thujene	1.44	918	924	29	iso‐3‐Thujanol	0.04	1136	—
9	α‐Pinene	1.19	925	939	30	Camphor	2.85	1146	1145
10	Camphene	0.43	941	954	31	β‐Pinene oxide	4.42	1163	—
11	Sabinene	3.12	969	975	32	Thujanol	0.48	1168	—
12	β‐Pinene	0.22	972	979	33	α‐Terpineol	0.88	1192	1190
13	Myrcene	0.14	989	991	34	Dihydrocarveol	0.06	1197	—
14	α‐Phellandrene	0.03	1003	1005	35	Safranal	0.01	1203	1202
15	Isoamyl isobutyrate	0.02	1012	—	36	cis‐3‐Hexenyl 2‐methylbutanoate	0.25	1233	—
16	α‐Terpinene	0.36	1016	1014	37	trans‐Myrtanol	0.04	1257	—
17	*p*‐Cymene	0.20	1024	1025	38	2‐(1‐Propenyl)phenol	0.09	1258	—
18	*o*‐Cymene	0.45	1026	1026	39	cis‐Verbenyl acetate	15.05	1277	1277
19	Sylvestrene	0.20	1028	—	40	neo‐iso‐3‐Thujyl acetate	0.09	1287	—
20	Eucalyptol (1,8‐Cineole)	10.71	1031	1033	41	Isobornyl acetate	0.21	1290	1289
21	Decahydronaphthalene	0.03	1054	—	42	Lavandulyl acetate	0.32	1293	—
**Total identified components** **96.28**									

*Note*: RI: Retention indices (experimental and literature) determined on a DB‐5MS Column. Reference values from Wiley/NIST databases. Entries Marked “–” are not available or not listed with verified values in the databases.

The identified compounds were mainly classified into monoterpene hydrocarbons, oxygenated monoterpenes, and ester derivatives. Oxygenated monoterpenes constituted the dominant fraction, followed by monoterpene hydrocarbons and ester compounds. Such chemical distribution is typical of *O. majorana* essential oils reported in previous phytochemical studies [41, 42].

Variations in essential oil composition are strongly influenced by geographical origin, climatic conditions, soil composition, and harvest season. The relatively high proportion of santolina triene (16.42%) observed in the present study may reflect a specific chemotype associated with the ecological conditions of the Tebessa region. Similar compositional variations have been reported for *O. majorana* essential oils collected from different Mediterranean areas.

### 3.2 In Vivo Antidiabetic Activities

#### 3.2.1 Hematological Parameters

Hematological analysis was conducted to assess systemic physiological alterations associated with diabetes and EO treatment. Since acarbose primarily acts as an intestinal α‐glucosidase inhibitor and does not directly influence hematological parameters, this group was not included in the hematological comparison.

The in vivo antidiabetic activity of *O. majorana* EO was evaluated through hematological parameters in alloxan‐induced diabetic rats. Alloxan, a well‐known diabetogenic agent, selectively destroys pancreatic β‐cells via oxidative stress, leading to persistent diabetes mellitus while exerting minimal effects on other cellular components [43].

The hematological profiles of the experimental groups are summarized in Table 2. The diabetic group exhibited a 44.4% decrease in lymphocyte count (LYM) (2.46 ± 0.5 × 10^9^/L) compared to the control group (4.43 ± 0.51 × 10^9^/L) (*p* < 0.001). Granulocyte count (GRA) also showed a 61.5% reduction in diabetic rats (1.5 ± 0.5 × 10^9^/L) compared to the control (3.9 ± 0.1 × 10^9^/L) (*p* < 0.05). Platelet count (PLT) decreased significantly by 48.4%, dropping from 919.6 ± 26.2 × 10^9^/L in the control group to 475.0 ± 25 × 10^9^/L in diabetic rats (*p* < 0.01).

**TABLE 2:** Plasma concentration of hematological parameters in different experimental groups.

	WBC(×10^9^/L)	LYM(×10^9^/L)	GRA(×10^9^/L)	HGB (g/dL)	RBC(×10^12^/L)	PLT (×10^9^/L)
Control	6.81 ± 0.73	4.43 ± 0.51	3.9 ± 0.1	11.93 ± 0.37	7.19 ± 0.06	919.6 ± 26.2
Diabetic	4.06 ± 1^NS^	2.46 ± 0.5^c^	1.5 ± 0.5^a^	12.46 ± 0.45^NS^	7.52 ± 0.05^b^	475.0 ± 25 ^b^
Diabetic+ EO	4.5 ± 0.41^NS^	3.36 ± 0.85^NS^	2.03 ± 0.95^NS^	12.56 ± 1.34 ^NS^	7.7 ± 0.56 ^NS^	912.6 ± 11.3^NS***^
EO	7.6 ± 0.52^NS^	4.8 ± 0.72 ^NS^	2.26 ± 0.64^NS^	10.96 ± 0.4 ^NS^	6.75 ± 0.25^NS^	772.3 ± 4.5 ^b^

*Note*: NS: Non‐significant differences; Comparison with the control group: *p* < 0.05 (a), *p* < 0.01 (b), *p* < 0.001 (c); Comparison with the diabetic group: *p* < 0.05 (*), *p* < 0.01 (**), *p* < 0.001 (***). Values are expressed as mean ± SD (*n* = 5). Different superscript letters indicate statistically significant differences.

Abbreviations: GRA, granulocytes; HGB, hemoglobin; LYM, lymphocytes; PLT, platelets; RBC, red blood cells; WBC, white blood cells

However, treatment with *O. majorana* EO significantly improved platelet levels, restoring them to 912.6 ± 11.3 × 10^9^/L (*p* < 0.001 compared to the diabetic group), indicating a protective effect against alloxan‐induced hematological alterations. The lymphocyte and granulocyte counts also increased in the EO‐treated diabetic group, reaching 3.36 ± 0.85 × 10^9^/L and 2.03 ± 0.95 × 10^9^/L, respectively, suggesting an immunoprotective role of the EO.

Lymphocytes and granulocytes are essential for immune defense as phagocytes that eliminate pathogens, but in uncontrolled diabetes, persistent hyperglycemia induces oxidative stress, leading to membrane protein oxidation and a decline in white blood cell (WBC) counts. This oxidative damage triggers lipid peroxidation, leukolysis, and weakened immune responses, as observed by a 40.4% reduction in WBC count in the diabetic group compared to the control group. Platelet abnormalities also pose significant clinical risks, with thrombocytopenia increasing bleeding risk and thrombocytosis leading to thrombotic events. Insulin regulates platelet function, and the absence of insulin in diabetic rats due to alloxan‐induced β‐cell destruction resulted in a 48.4% decrease in platelet count. However, treatment with *O. majorana* essential oil restored platelet levels, suggesting a protective effect against diabetes‐induced hematological disruptions [44, 45].

#### 3.2.2 Glycemia and Liver Function Parameters

The results revealed a significant increase in fasting blood glucose (FBS) and liver enzyme levels (AST, ALT, and ALP) in the diabetic group, reinforcing the notion that diabetes mellitus is a metabolic disorder marked by persistent hyperglycemia due to insulin deficiency or resistance. The primary mechanism driving hyperglycemia in diabetes involves excessive glucose synthesis and impaired glucose uptake by tissues [46].

Liver enzymes such as aspartate aminotransferase (AST) and alanine aminotransferase (ALT) are cytosolic enzymes, whereas alkaline phosphatase (ALP) is a membrane‐bound enzyme. These enzymes are predominantly localized in liver cells and are released into the bloodstream in significant amounts only when hepatocellular damage occurs, leading to membrane leakage or rupture. An elevation in serum levels of these enzymes serves as a biochemical indicator of liver dysfunction [47].

As presented in Table 3, the diabetic group exhibited a marked increase in blood glucose levels (1.47 ± 0.04 g/L) compared to the control group (0.65 ± 0.05 g/L). However, treatment with *O. majorana* EO (Diabetic + EO group) resulted in a notable reduction in blood glucose (0.94 ± 0.03 g/L, *p* < 0.01), suggesting a potential hypoglycemic effect of the EO. Similarly, the reference drug acarbose effectively reduced blood glucose to near‐control levels (0.74 ± 0.04 g/L, *p* < 0.01).

**TABLE 3:** Concentration of glucose and parameters of liver function of different experimental groups.

	FBS (g/L)	AST (U/L)	ALT (U/L)	ALP (IU/L)
Control	0.65 ± 0.05	242.3 ± 0.57	85.3 ± 3.05	370.6 ± 11
Diabetic	1.47 ± 0.04^c^	310.3 ± 21.9^a^	127.0 ± 7^a^	843.3 ± 3.5^c^
Diabetic+ acarbose	0.74 ± 0.04^NS**^	315.6 ± 14.2^a NS^	88 ± 2 ^NS**^	319.6 ± 70.5^NS**^
Diabetic+ EO	0.94 ± 0.03^a**^	242.0 ± 2^NS*^	76.0 ± 11.53 ^NS*^	842.6 ± 2.51^c NS^
EO	0.96 ± 0.01^b^	216.0 ± 6^a^	71.0 ± 4^a^	241 ± 1^b^

*Note*: NS: Non‐significant differences; Comparison with the control group: *p* < 0.05 (a), *p* < 0.01 (b), *p* < 0.001 (c); Comparison with the diabetic group: *p* < 0.05 (*), *p* < 0.01 (**), *p* < 0.001 (***). Values are expressed as mean ± SD (*n* = 5). Different superscript letters indicate statistically significant differences.

Abbreviations: GRA, granulocytes; HGB, hemoglobin; LYM, lymphocytes; PLT, platelets; RBC, red blood cells; WBC, white blood cells

Regarding liver function parameters, the diabetic group showed a significant elevation in AST (310.3 ± 21.9 U/L), ALT (127.0 ± 7 U/L), and ALP (843.3 ± 3.5 IU/L) levels compared to the control group (242.3 ± 0.57 U/L, 85.3 ± 3.05 U/L, and 370.6 ± 11 IU/L, respectively), indicating liver damage. Treatment with *O. majorana* EO restored AST (242.0 ± 2 U/L, *p* < 0.05) and ALT (76.0 ± 11.53 U/L, *p* < 0.05) to near‐control levels, suggesting a hepatoprotective effect. However, ALP levels in the EO‐treated diabetic group (842.6 ± 2.51 IU/L) remained significantly elevated, similar to the diabetic group, indicating that EO may not fully mitigate diabetes‐induced hepatic dysfunction.

Acarbose treatment exhibited a similar hepatoprotective effect, with AST (315.6 ± 14.2 U/L) and ALT (88 ± 2 U/L) levels showing no significant differences compared to the control group. Additionally, ALP levels in the acarbose‐treated group (319.6 ± 70.5 IU/L) were significantly reduced compared to the diabetic group, demonstrating its efficacy in preventing diabetes‐associated liver damage.

Interestingly, the EO‐only group did not show significant alterations in glucose or liver enzyme levels, with FBS (0.96 ± 0.01 g/L), AST (216.0 ± 6 U/L), ALT (71.0 ± 4 U/L), and ALP (241 ± 1 IU/L) remaining within the normal range, reinforcing its safety profile.

#### 3.2.3 Kidney Function Parameters and Triglycerides

Diabetes‐induced hyperglycemia is associated with significant elevations in serum urea, creatinine (CT), and uric acid (UA), which are considered key markers of kidney dysfunction [48]. As presented in Table 4, the diabetic group exhibited a significant increase in key kidney function markers, including serum urea (0.39 ± 0.03 g/L, *p* < 0.05), CT (4 ± 0.0 mg/L, *p* < 0.05), and UA (21 ± 1 mg/L, *p* < 0.05), compared to the control group (0.2 ± 0.04 g/L, 3 ± 0.0 mg/L, and 14.67 ± 1.52 mg/L, respectively). This elevation indicates impaired kidney function likely resulting from diabetes‐induced oxidative stress and renal damage.

**TABLE 4:** Parameters of kidney function and triglycerides of different experimental groups.

	Urea (g/L)	CT (mg/L)	UA (mg/L)	TG (g/L)
Control	0.2 ± 0.04	3 ± 0.0	14.67 ± 1.52	0.37 ± 0.02
Diabetic	0.39 ± 0.03^a^	4 ± 0.0^a^	21 ± 1^a^	1.03 ± 0.03^c^
Diabetic+ acarbose	0.39 ± 0.027^NS^	3 ± 0.0^NS^	16.67 ± 5.5 ^NS^	0.54 ± 0.07^NS**^
Diabetic+ EO	0.27 ± 0.02^NS**^	3 ± 0.0^NS^	18 ± 0.0 ^NS*^	0.54 ± 0.04^b***^
EO	0.27 ± 0.02^NS^	3 ± 0.0 ^NS^	18.0 ± 1^a^	0.57 ± 0.02^NS^

*Note*: Comparison with diabetic group: * *p* <0.05; ** *p* <0.01; *** *p* <0.001, comparison with control group: ^a^ *p* <0.05; ^b^ *p* <0.01; c*p* <0.001, (NS) non significative. Values are expressed as mean ± SD (*n* = 5). Different superscript letters indicate statistically significant differences.

Abbreviations: GRA, granulocytes; HGB, hemoglobin; LYM, lymphocytes; PLT, platelets; RBC, red blood cells; WBC, white blood cells

Treatment with *O. majorana* EO (Diabetic + EO group) led to a significant reduction in serum urea levels (0.27 ± 0.02 g/L, *p* < 0.01) compared to the diabetic group, suggesting a protective effect on kidney function. Similarly, UA levels showed a modest decrease (18 ± 0.0 mg/L, *p* < 0.05), while CT levels (3 ± 0.0 mg/L) remained unchanged. This indicates that EO treatment may partially alleviate diabetes‐induced renal dysfunction.

Acarbose treatment (diabetic + acarbose group) also showed a protective effect, as serum urea (0.39 ± 0.027 g/L) and CT (3 ± 0.0 mg/L) levels were comparable to the diabetic group, whereas UA levels (16.67 ± 5.5 mg/L) were restored closer to normal, albeit without statistical significance.

Regarding lipid metabolism, the diabetic group exhibited a significant elevation in TG levels (1.03 ± 0.03 g/L, *p* < 0.001) compared to the control group (0.37 ± 0.02 g/L), which is a hallmark of diabetic dyslipidemia. Treatment with *O. majorana* EO significantly reduced TG levels (0.54 ± 0.04 g/L, *p* < 0.001), highlighting its potential lipid‐lowering effect. Similarly, acarbose‐treated diabetic rats showed a significant decrease in TG levels (0.54 ± 0.07 g/L, *p* < 0.01), reinforcing its role in improving lipid metabolism.

Interestingly, the EO‐only group did not exhibit significant alterations in kidney function markers, with urea (0.27 ± 0.02 g/L), CT (3 ± 0.0 mg/L), and UA (18 ± 1 mg/L) remaining within a normal range. However, TG levels (0.57 ± 0.02 g/L) were slightly elevated compared to the control group, though not significantly different from the diabetic + EO group. This suggests that *O. majorana* EO may have a modulatory effect on lipid metabolism while maintaining renal function.

#### 3.2.4 Histopathological Studies

**FIGURE 1:**
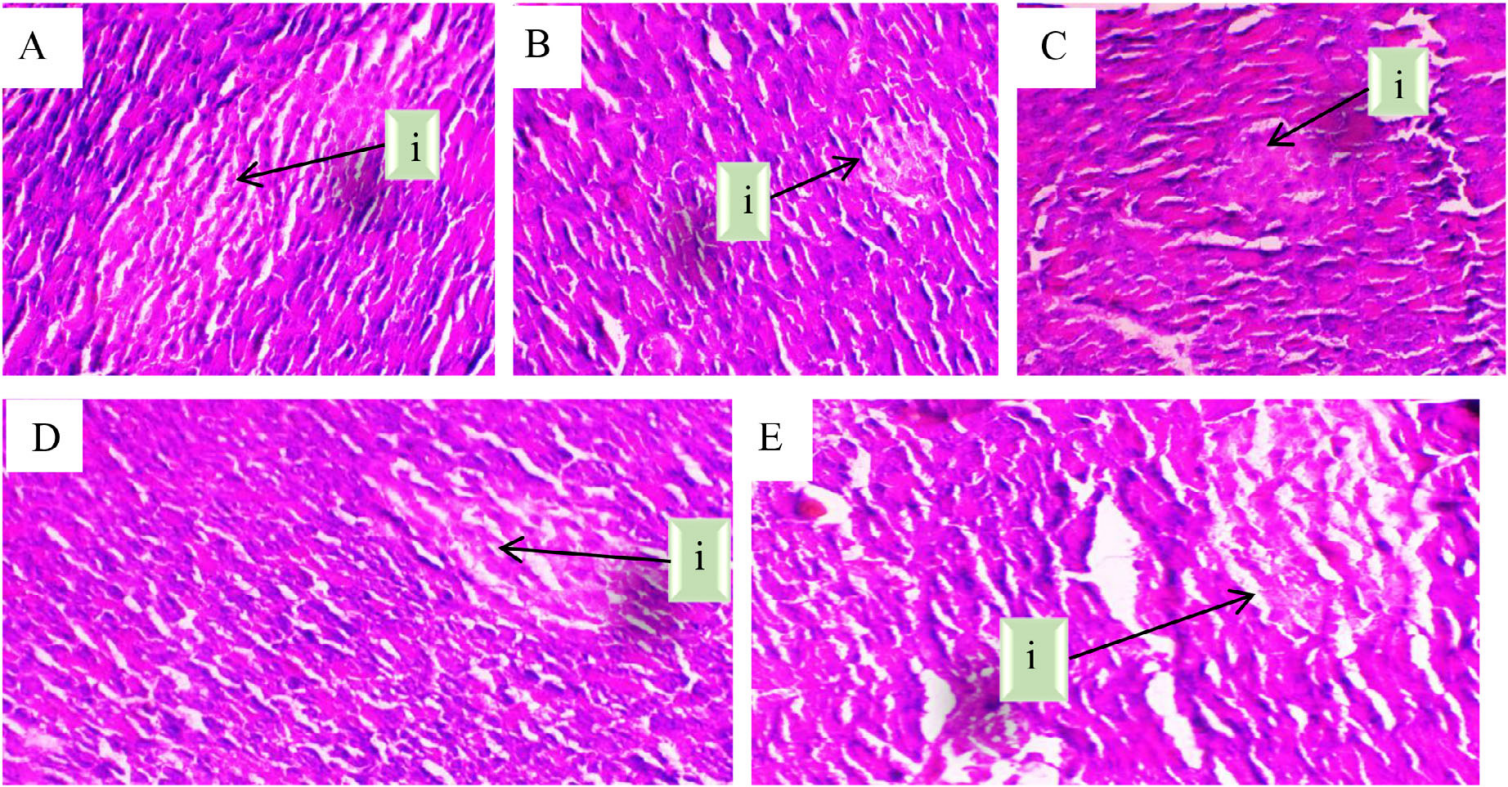
Microscopic observation of pancreatic histological sections from different experimental groups, (A) Control group, (B) Diabetic group, (C) Diabetic + acarbose group, (D) Diabetic + EO group, and (E) EO‐only group. (i) Indicates the islets of Langerhans. Magnification ×40.

These histopathological findings further support the biochemical results, demonstrating the protective effects of *O. majorana* EO against alloxan‐induced pancreatic damage. The diabetic group exhibited extensive structural deterioration, with irregularly shaped and shrunken islets of Langerhans, a degraded connective tissue layer, and a marked reduction in β‐cells (Figure 1). This suggests severe pancreatic dysfunction, aligning with the observed hyperglycemia and reduced insulin regulation in the diabetic state. Interestingly, the acarbose‐treated group showed only partial protection, as the histological features remained largely similar to those of the diabetic group, with persistent islet shrinkage and β‐cell loss (Figure 1). This indicates that while acarbose effectively reduced glycemia, it had limited regenerative effects on pancreatic tissue.

In contrast, EO treatment significantly improved pancreatic morphology, as evidenced by the restoration of islet structure, an increase in β‐cell number, and well‐preserved connective tissue (Figure 1). This suggests that EO not only lowers blood glucose levels but may also exert protective or protective effects on pancreatic β‐cells. The observed structural recovery could be attributed to the antioxidant and anti‐inflammatory properties of EO, which may counteract alloxan‐induced oxidative stress and promote β‐cell survival. Overall, these findings highlight the potential of *O. majorana* EO as a promising natural agent for managing diabetes, with both glycemic control and pancreatic protective effects.

In addition to qualitative observations, pancreatic damage was semi‐quantitatively evaluated using a histological scoring system assessing islet architecture disruption, β‐cell degeneration, and inflammatory infiltration.

### 3.3 In Vitro Antidiabetic Activities

To further elucidate the potential mechanism underlying the observed hypoglycemic effects in vivo, the inhibitory activity of *O. majorana* EO against key carbohydrate‐digesting enzymes was investigated in vitro.

#### 3.3.1 Enzymatic Inhibition of α‐Amylase and α‐Glucosidase

**FIGURE 2:**
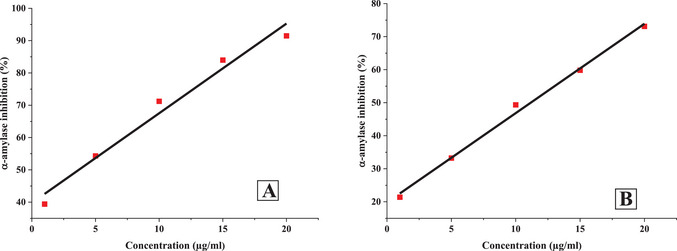
Linear regression of 𝛼‐amylase inhibition at different concentration of *O. majorana* (A), and acarbose (B).

**FIGURE 3:**
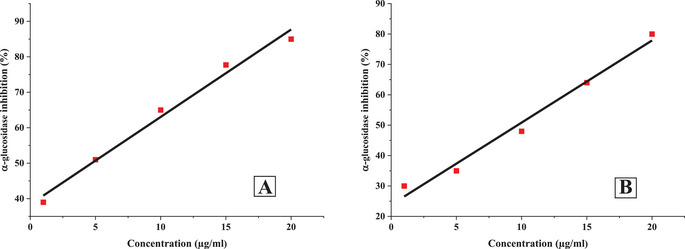
Linear regression of 𝛼‐glucosidase inhibition at different concentration of *O. majorana* (A), and acarbose (B).

The inhibitory potential of *O. majorana* EO and acarbose against α‐amylase and α‐glucosidase was found to be dose‐dependent, with concentrations ranging from 1 to 20 µg/mL (Figures 2 and 3). As the concentration increased, a significant reduction in enzymatic activity was observed.

At the highest tested concentration (20 µg/mL), acarbose exhibited significant inhibition, reducing α‐amylase and α‐glucosidase activities by 73.11% and 80.2%, respectively. Similarly, *O. majorana* EO demonstrated a comparable inhibitory effect, achieving 91.49% inhibition for α‐amylase and 85.15% for α‐glucosidase at the same concentration.

To further evaluate the inhibitory potential of *O. majorana* EO, the IC_50_ values for α‐amylase and α‐glucosidase inhibition were determined and compared to those of acarbose (Table 5).

**TABLE 5:** IC_50_ values of *O. majorana* EO and acarbose for α‐amylase and α‐glucosidase inhibition.

		Equation	*R* ^2^	IC_50_ (ug/mL)
α‐amylase	Acarbose	I%=19.809+2.704×[C]	0.995	11.17
	*O. Majorana*	I%=39.777+2.775×[C]	0.975	3.68
α‐glucosidase	Acarbose	I%=23.841+2.702×[C]	0.982	9.68
	*O. Majorana*	I%=38.395+2.465×[C]	0.986	4.71

IC_50_ values were calculated from concentration–response curves using linear regression analysis, and the reliability of the models was confirmed by high correlation coefficients (*R* ^2^ > 0.975).

The results revealed that *O. majorana* EO exhibited stronger inhibitory activity, with lower IC_50_ values for both enzymes. Specifically, the IC_50_ for α‐amylase inhibition was 3.68 µg/mL, significantly lower than acarbose (11.17 µg/mL). Similarly, for α‐glucosidase inhibition, the EO showed an IC_50_ of 4.71 µg/mL, whereas acarbose had an IC_50_ of 9.68 µg/mL. These findings indicate that *O. majorana* EO possesses a potent inhibitory effect on key carbohydrate‐digesting enzymes, suggesting its potential as a natural alternative for controlling postprandial hyperglycemia.

The regression equations further support this observation, with *R* ^2^> 0.975 exceeding 0.975, indicating a strong correlation between EO concentration and enzymatic inhibition. The regression model for *O. majorana* EO demonstrated higher inhibitory efficiency, as reflected in the steeper slopes of its equations compared to acarbose. This suggests that even at lower concentrations, the EO exhibits substantial inhibition of both α‐amylase and α‐glucosidase.

The superior inhibitory activity of *O. majorana* EO may be attributed to its bioactive compounds, which likely interfere with enzyme active sites, preventing the breakdown of carbohydrates into glucose. This mechanism plays a crucial role in managing postprandial blood sugar levels, reinforcing the potential of EO as a natural therapeutic agent for diabetes control.

#### 3.3.2 Binding Constants

The interaction between α‐amylase or α‐glucosidase and the tested inhibitors (*O. majorana* EO and acarbose) was evaluated by monitoring absorbance variations with increasing concentrations of EO or acarbose. A progressive decrease in absorption values of the enzyme solutions indicates the formation of stable enzyme‐inhibitor complexes. This suggests that EO components effectively bind to the active sites of α‐amylase and α‐glucosidase, thereby reducing their catalytic activity.

To quantify these interactions, the binding constant (K_b_) and binding free energy (ΔG) were calculated using Equations (3) and (4) [49].

(3) $$\frac{A}{A_{0}-A}=\frac{\varepsilon }{\varepsilon_{0}-\varepsilon }+\frac{\varepsilon }{\varepsilon_{0}-\varepsilon }\frac{1}{K_{b}\times C}$$

(4) $$\Delta G=-nRT\ln K_{b}$$

where A_0_ and A represent the absorbance values of α‐amylase or α‐glucosidase and their complexes with EO or acarbose, respectively, $\varepsilon_{0}$ and ε are the extinction coefficients of the free and bound enzyme, respectively, C is the concentration of EO or acarbose, R is the gas constant (8.32 J.mol^−1^K^−1^) and T is ambient temperature (298K).

The calculated K_b_ and their corresponding ΔG further confirm the strong interactions between *O. majorana* EO, acarbose, and the target enzymes (Table 6). The regression equations demonstrate high correlation coefficients (*R* ^2^> 0.988), reinforcing the reliability of the binding data.

**TABLE 6:** Binding constant and binding energy values for the interaction of *O. majorana* EO, and acarbose with α‐amylase and α‐glucosidase.

		Equation	*R* ^2^	K_b_ (M^−1^)	ΔG (KJ.mol^−1^)
α‐amylase	Acarbose	$\;\frac{A}{A_{0}-A}=-0.918-0.016\frac{1}{\left[C\right]}$	0.992	3.62 × 10^4^	−26.02
	*O. Majorana*	$\;\frac{A}{A_{0}-A}=-0.869-0.032\frac{1}{\left[C\right]}$	0.992	4.03 × 10^4^	−26.12
α‐glucosidase	Acarbose	$\;\frac{A}{A_{0}-A}=-0.711-0.018\frac{1}{\left[C\right]}$	0.990	2.49 × 10^4^	−25.10
	*O. Majorana*	$\;\frac{A}{A_{0}-A}=-0.816-0.037\frac{1}{\left[C\right]}$	0.988	3.35 × 10^4^	−25.83

Notably, *O. majorana* EO exhibited a higher binding affinity for both α‐amylase (K_b_ = 4.03 × 10^4^ M^−1^) and α‐glucosidase (K_b_ = 3.35× 10^4^ M^−1^) compared to acarbose (K_b_ = 3.62 × 10^4^ M^−1^ and 2.49 × 10^4^ M^−1^, respectively). This suggests that EO forms more stable enzyme‐inhibitor complexes, leading to stronger inhibitory effects.

Additionally, the negative ΔG values indicate that the binding interactions are spontaneous, with *O. majorana* EO exhibiting slightly more favorable binding energy (−26.12 kJ/mol for α‐amylase and −25.83 kJ/mol for α‐glucosidase) than acarbose. Along with the in vivo results, these findings suggest that *O. majorana* EO effectively inhibits carbohydrate‐digesting enzymes, contributing to improved glycemic control and supporting its potential as a natural anti‐diabetic agent.

### 3.4 In Silico Antidiabetic Activities

#### 3.4.1 Molecular Docking

**FIGURE 4:**
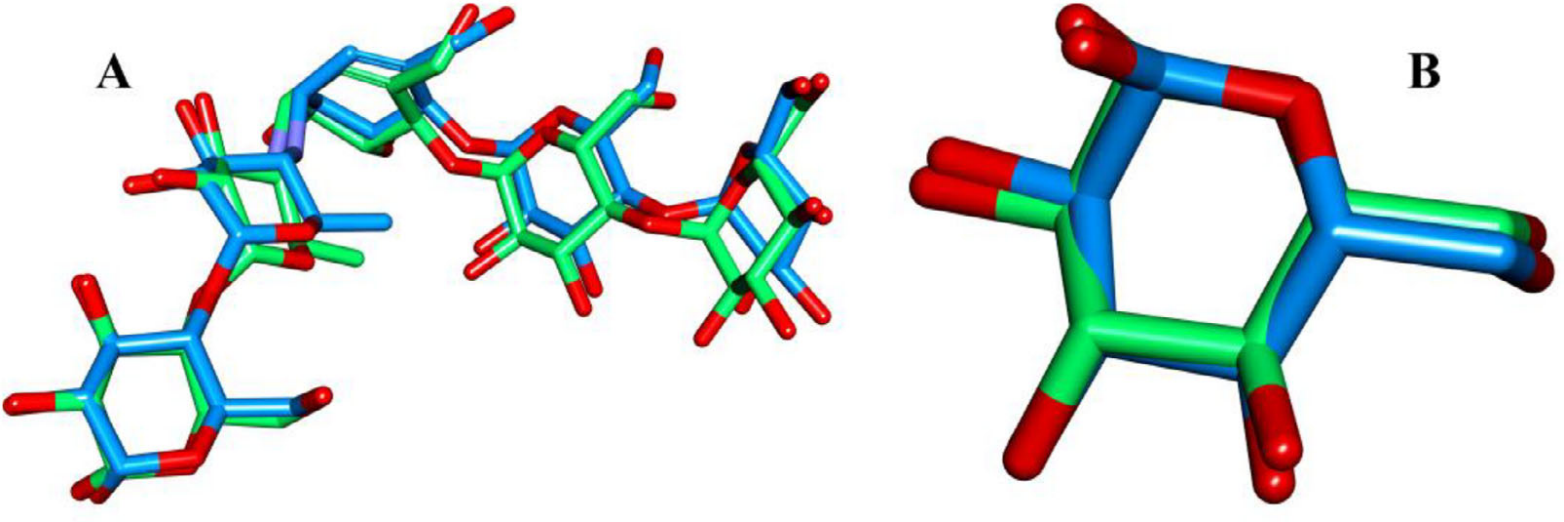
(Green) native ligand (Bleu) dock pose in the active site of A:3BAJ and B:3A4A.

The docking protocol was validated by re‐docking the co‐crystallized ligands into their respective binding sites. The obtained RMSD values were 0.813 Å for α‐amylase and 0.350 Å for α‐glucosidase (Figure 4), indicating strong agreement between the docked and native ligand conformations. These low RMSD values confirm the accuracy of the docking model in reproducing experimental binding poses, ensuring its reliability for predicting the interactions of *O. majorana* EO compounds with the target enzymes.

The molecular docking results revealed negative binding affinities for all tested compounds against both α‐amylase and α‐glucosidase, indicating their potential inhibitory interactions with these enzymes. To focus on the most biologically relevant interactions, only major compounds with abundances exceeding 1% were analyzed, as they are more likely to contribute significantly to the overall enzymatic inhibition.

The reference drug, acarbose, exhibited ΔG values of −4.51 kcal/mol and −6.09 kcal/mol with α‐amylase and α‐glucosidase, respectively. Among the most abundant *O. majorana* EO constituents, trans‐thujone (33.30%), cis‐verbenyl acetate (15.05%), eucalyptol (10.71%), β‐pinene oxide (4.42%), and camphor (2.85%) displayed strong binding affinities, surpassing acarbose for both enzymes. Their ΔG values ranged from −6.64 to −5.19 kcal/mol for α‐amylase and −7.27 to −6.24 kcal/mol for α‐glucosidase (Table 7).

**TABLE 7:** Binding energy (kcal/mol) of abundant *O. majorana* EO compounds.

	3BAJ	3A4A		3BAJ	3A4A
Native ligand	−11.88	−6.39	Acarbose	−4.51	−6.09
α‐pinene	−4.81	−5.81	Eucalyptol	−5.19	−6.34
α‐Thujene	−4.73	−5.56	Sabinene	−4.80	−5.66
β‐pinene oxide	−5.40	−6.46	Santolina triene	−4.27	−5.05
Camphor	−5.23	−6.60	Trans‐thujone	−5.64	−6.24
Cis‐verbenylacetate	−6.64	−7.27			

Notably, cis‐verbenyl acetate (−6.64 kcal/mol), trans‐thujone (−5.64 kcal/mol), β‐pinene oxide (−5.40 kcal/mol) camphor (−5.23 kcal/mol)and eucalyptol (−5.19 kcal/mol) exhibited the highest affinities for α‐amylase. Similarly, cis‐verbenyl acetate (−7.27 kcal/mol), camphor (−6.60 kcal/mol), β‐pinene oxide (−6.46 kcal/mol), eucalyptol (−6.34 kcal/mol), and trans‐thujone (−6.24 kcal/mol) showed the strongest interactions with α‐glucosidase. These results suggest that these major EO constituents are responsible for the observed inhibitory activity, supporting their role as key contributors to the enzyme inhibition potential of *O. majorana* EO.

**FIGURE 5:**
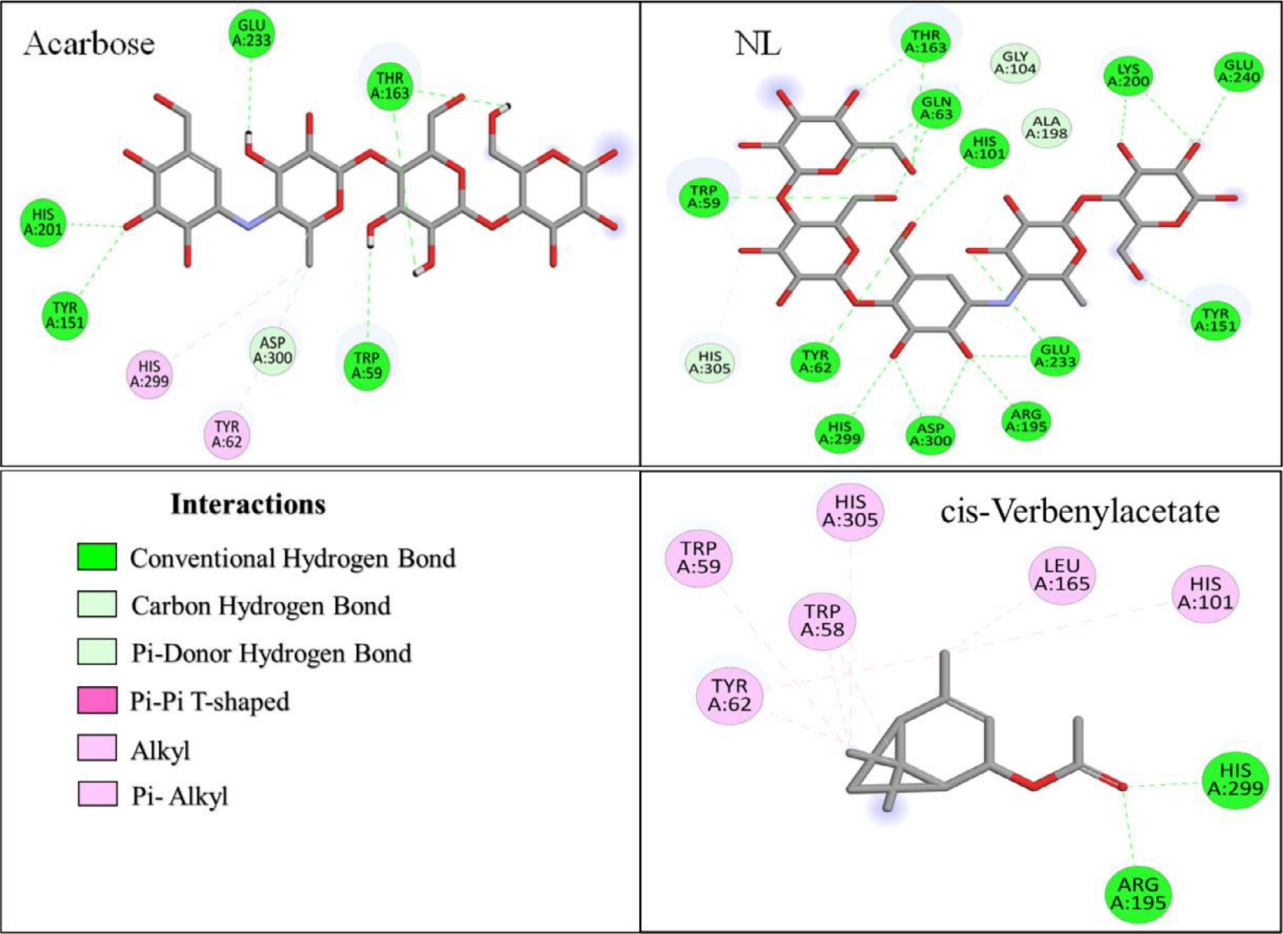
2D Binding interactions of selected *O. majorana* EO compounds, NL and acarbose with the active site of α‐amylase.

As shown in Figure 5, the native ligand (NL) of α‐amylase was stabilized through interactions with fifteen amino acid residues, forming t26 hydrogen bonds (H‐bonds). Key interacting residues included TRP A:59, TYR A:62, GLN A:63, HIS A:101, GLY A:104, TYR A:151, THR A:163, ARG A:195, ALA A:198, LYS A:200, GLU A:233, GLU A:240, HIS A:299, ASP A:300, and HIS A:305. In comparison, the reference drug ARE interacted with eight residues, seven of which overlapped with those of NL, forming seven H‐bonds and two hydrophobic interactions, highlighting its strong binding affinity.

Among the most potent EO compounds, cis‐verbenyl acetate displayed extensive interactions, engaging with nine residues (Figure 5), including TRP A:58, TRP A:59, TYR A:62, THR A:163, LEU A:165, ARG A:195, HIS A:101, HIS A:299, and HIS A:305. It was stabilized by three H‐bonds and eight hydrophobic interactions, suggesting a strong affinity for the enzyme's active site. Trans‐thujone and β‐pinene oxide exhibited a more selective binding pattern, interacting with four and five residues, respectively (Figure S1). Trans‐thujone formed three H‐bonds with ARG A:195 and HIS A:299 and three hydrophobic interactions with TRP A:58 and TYR A:62, while β‐pinene oxide established two H‐bonds with ASP A:197 and ASP A:300, and five hydrophobic interactions with TRP A:58, TYR A:62 and HIS A:305, which are crucial for enzyme activity.

Additionally, camphor and eucalyptol displayed moderate interactions with α‐amylase, engaging with five and four residues, respectively (Figure S1). Camphor was stabilized through six H‐bonds and three hydrophobic interactions, primarily involving TYR A:62, TRP A:59, and HIS A:101. In contrast, eucalyptol primarily formed seven hydrophobic interactions, particularly with TRP A:59 and TYR A:62, indicating a distinct stabilization mechanism compared to other compounds.

**FIGURE 6:**
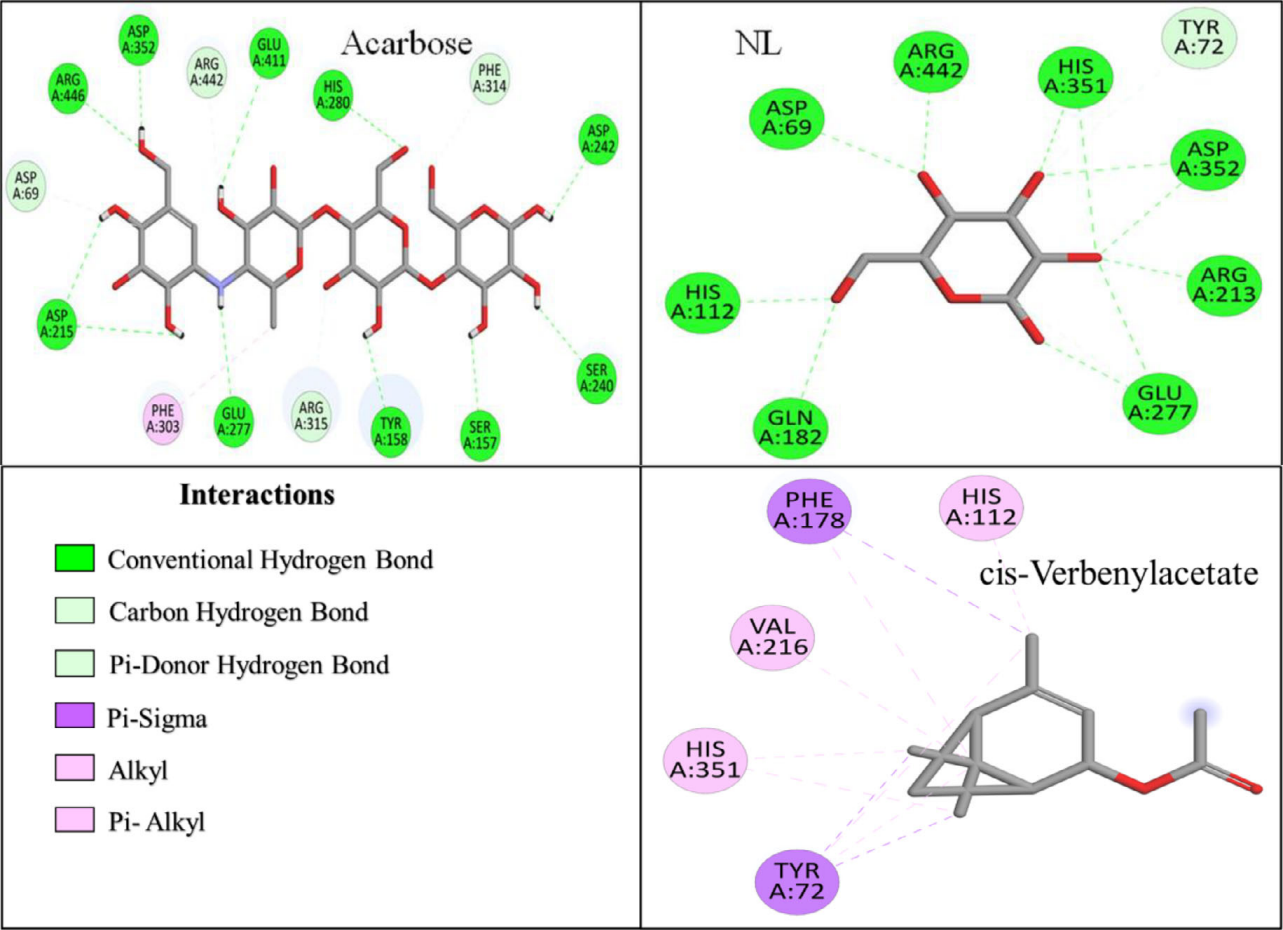
2D Binding interactions of selected *O. majorana* EO compounds, NL and acarbose with the active site of α‐glucosidase.

As illustrated in Figure 6, the NL of α‐glucosidase was stabilized by interactions with nine amino acid residues, forming a total of fifteen H‐bonds. The key interacting residues included ASP A:69, TYR A:72, HIS A:112, GLN A:182, ARG A:213, GLU A:277, HIS A:351, ASP A:352, and ARG A:442. In comparison, ARE exhibited strong binding affinity, interacting with fifteen residues, forming sixteen H‐bonds with ASP A:69, SER A:157, TYR A:158, ASP A:215, SER A:240, ASP A:242, GLU A:277, HIS A:280, PHE A:303, PHE A:314, ARG A:315, ASP A:352, GLU A:411, ARG A:442, and ARG A:446 (Figure 6).

All the EO compounds showed hydrophobic interactions with governing residues such as TYR A:72, HIS A:112, VAL A:216, HIS A:351, and PHE A:178, and exhibited common binding tendencies (Figures 6 and S2). Among all the EO compounds evaluated, cis‐verbenyl acetate exhibited the strongest interaction, which occurred with five residues, including TYR A:72, HIS A:112, and HIS A:351. It was stabilized through 10 hydrophobic interactions, which indicated its excellent binding mode.

Trans‐thujone, β‐pinene oxide, camphor, and eucalyptol were found to possess moderate binding affinities, interacting with six, five, seven, and four residues, respectively (Figure S2). Trans‐thujone formed three H‐bonds and six hydrophobic interactions, prominently with TYR A:72, PHE A:178, ARG A:213, VAL A:216 and HIS A:351, while β‐pinene oxide formed two H‐bonds and five hydrophobic interactions, mainly with TYR A:72, PHE A:159, PHE A:178, VAL A:216, ARG A:442. Camphor exhibited a distinct binding mode, forming three H‐bonds and seven hydrophobic interactions, stabilizing within the active site via interactions with TYR A:72, HIS A:112, PHE A:159, PHE A:178, ARG A:213, VAL A:216, and HIS A:351. Eucalyptol, on the other hand, relied predominantly on ten hydrophobic interactions, engaging with TYR A:72, PHE A:178, VAL A:216, and HIS A:351 suggesting a different stabilization mechanism.

#### 3.4.2 ADMET Analysis

Assessment of the physicochemical properties of potential therapeutic compounds is a critical step in drug discovery, as they dictate pharmacokinetics, bioavailability, and toxicity, hence their efficacy and safety in vivo studies [50]. ADMET analysis provides valuable insights into the pharmacokinetic profile, drug‐likeness, and potential toxicity of potential compounds, which aids in evaluating their therapeutic potential [51].

In this study, the five best‐performing EO compounds from molecular docking, cis‐verbenyl acetate (CVA), β‐pinene oxide (BPO), camphor (CMP), trans‐thujone (TTJ), and eucalyptol (EUC), were evaluated based on Lipinski's ‘rule of five’, as well as the Ghose, Veber, Egan, and Muegge drug‐likeness criteria (Tables 8 and 9).

**TABLE 8:** Pharmacokinetic and physicochemical properties of the top compounds.

	ARE	CVA	BPO	CMP	TTJ	EUC
MW (g/mol)	645.60	194.27	152.23	152.23	152.23	154.25
Num HBA	19	2	1	1	1	1
Num HBD	14	0	0	0	0	0
Consensus LOGP	−6.26	2.75	2.42	2.37	2.35	2.67
TPSA (Å^2^)	329.01	26.30	44.70	17.07	17.07	9.23
LogS (ESOL)	2.57	−3.26	−2.08	−2.16	−2.15	−2.52
GI absorption	Low	High	High	High	High	High
p‐gp substrat	Yes	No	No	No	No	No
CYP3A4	No	No	No	No	No	No
Log Kp (cm/s)	−16.50	−4.84	−5.77	−5.67	−5.62	−5.30
SA score	7.25	4.50	4.06	3.22	2.79	3.65

**TABLE 9:** Druglikeness parameters of the top compounds.

	ARE	CVA	BPO	CMP	TTJ	EUC
Lipinski	No 3V	Yes 0V	Yes 0V	Yes 0V	Yes 0V	Yes 0V
Ghose	No 4V	Yes	No 1V	No 1V	No 1V	No 1 V
Veber	No 2V	Yes	Yes	Yes	Yes	Yes
Egan	No 1V	Yes	Yes	Yes	Yes	Yes
Muegge	No 5V	No 1V	No 2V	No 2 V	No 2V	No 2V
BAS	0.17	0.55	0.55	0.55	0.55	0.55

The Consensus LogP values of all the chosen compounds were less than 5, which showed good permeability through biological membranes. The total polar surface area (TPSA) ranged from 9.23 to 47.70 Å^2^, with hydrogen bond acceptors (HBA) < 10 and hydrogen bond donors (HBD) < 5, reflecting good oral bioavailability [52].

Pharmacokinetic analysis revealed high gastrointestinal (GI) absorption for all EO compounds, alongside high skin permeability (Log Kp values < ‐2.5) [53]. Notably, none of these compounds inhibited CYP3A4, a key enzyme in drug metabolism [54], implying efficient hepatic metabolism with a lower risk of metabolic inhibition‐related adverse effects.

Additionally, the synthetic accessibility (SA) scores of the EO compounds ranged from 2.79 to 4.50, indicating they are relatively easy to synthesize compared to the reference drug ARE (7.25). This suggests a potential advantage for EO‐derived compounds in terms of feasibility for large‐scale synthesis.

All selected compounds complied with Lipinski's, Veber's, and Egan's rules, indicating favorable oral bioavailability, whereas the reference drug ARE did not meet these criteria. Additionally, CVA fully adhered to Ghose's rules, while the other compounds exhibited one violation due to their molecular weights (MW) being below 160 g/mol (Table 8). Regarding Muegge's criteria, CVA showed only one violation (MW < 200), whereas the remaining compounds had additional violations related to low molecular weight (<200 g/mol) and insufficient heteroatoms (<2).

Moreover, all selected compounds demonstrated high bioavailability scores (BAS) of 0.55, which is significantly higher than ARE (0.17), suggesting a greater likelihood of biological activity [55].

The LD_50_ values for all EO compounds ranged from 500 to 5000 mg/kg, indicating varying degrees of acute toxicity. Among them, BPO (5000 mg/kg), CVA (2600 mg/kg), and EUC (2480 mg/kg) exhibited the highest LD50 values, classifying them under toxicity class 5 and suggesting low acute toxicity with safer toxicity profiles compared to the reference drug ARE (2000 mg/kg).

In contrast, CMP (775 mg/kg) and TTJ (500 mg/kg) showed the lowest LD50 values, indicating higher acute toxicity potential. Both TTJ, CMP, and ARE fall under Toxicity Class 4, suggesting a slightly higher toxicological risk compared to the other compounds (Table 10).

**TABLE 10:** Toxicity prediction of the top compounds.

	ARE	BPO	CVA	CMP	TTJ	EUC
LD_50_ (mg/kg)	2000	5000	2600	775	500	2480
Toxicity class	4	5	5	4	4	5
Hepatotoxicity	Act 50%	Inact 79%	Inact 53%	Inact 72%	Inact 76%	Inact 86%
Carcinogenicity	Inact 84%	Inact 51%	Inact 53%	Inact 63%	Inact 62%	Inact 68%
Immunotoxicity	Act 91%	Inact 91%	Act 78%	Inact 96%	Inact 99%	Inact 99%
Mutagenicity	Inact 76%	Inact 95%	Inact81 %	Inact94 %	Inact 87%	Inact 96%
Cytotoxicity	Inact 69%	Inact 76%	Inact 75%	Inact 61%	Inact 67%	Inact 75%

Abbreviations: Act, Active; Inact, Inactive

All compounds were predicted to be inactive for hepatotoxicity, carcinogenicity, mutagenicity, and cytotoxicity, implying a low risk of liver damage, cancer‐related effects, genetic mutations, and cellular toxicity. However, ARE exhibited hepatotoxic activity (50%), indicating potential liver toxicity risks. Furthermore, while most compounds were non‐immunotoxic, CVA and ARE displayed active immunotoxicity, suggesting possible effects on the immune system.

#### 3.4.3 MD Simulation Analysis

MD simulations provide crucial insights into the structural stability and dynamic behavior of biomolecular complexes under physiological conditions. Based on molecular docking results, BPO and CVA were selected for MD simulations in complex with α‐amylase and α‐glucosidase due to their highest binding free energy (ΔG) and LD50 values, indicating strong binding affinity and favorable toxicity profiles.

The stability and interaction dynamics of these protein‐ligand complexes were assessed by analyzing key structural parameters, including RMSD to evaluate overall stability, RMSF to determine residue‐level flexibility, Rg to assess the compactness of the protein structure, and SASA to investigate solvent exposure and molecular interactions.

Table 11 presents the average values of these parameters, offering a comprehensive evaluation of the structural variations and interaction dynamics throughout the MD simulations.

**TABLE 11:** Average values of RMSD, RMSF, Rg, and SASA obtained from MD simulations studies.

α‐amylase (3BAJ)				α‐glucosidase (3A4A)		
	ARE	CVA	BPO	ARE	CVA	BPO
RMSD (nm)	0.192	0.142	0.114	0.355	0.292	0.291
RMSF (nm)	0.115	0.111	0.112	0.119	0.150	0.109
SASA (nm^2^)	205.960	200.651	204.735	239.067	255.607	318.131
Rg (nm)	2.367	2.353	2.366	2.432	2.498	2.479

For the α‐amylase (3BAJ) complexes, the RMSD analysis showed that the 3BAJ‐BPO complex maintained the most stable structure during the simulation, with the lowest RMSD value (0.114 nm) compared to 3BAJ‐CVA complex (0.142 nm) and the reference complex (0.192 nm). In contrast, the reference complex exhibited higher RMSD values, indicating greater structural fluctuations and decreased stability over time. In addition, the RMSF analysis demonstrated that all systems exhibited nearly equivalent atomic flexibility, with average RMSF values falling within a narrow range of 0.111 to 0.115 nm. This consistency signifies that each complex's amino acid residues experienced comparable degrees of mobility, suggesting that their structural dynamics remained stable and consistent throughout the simulation, without significant deviations in flexibility or movement patterns.

**FIGURE 7:**
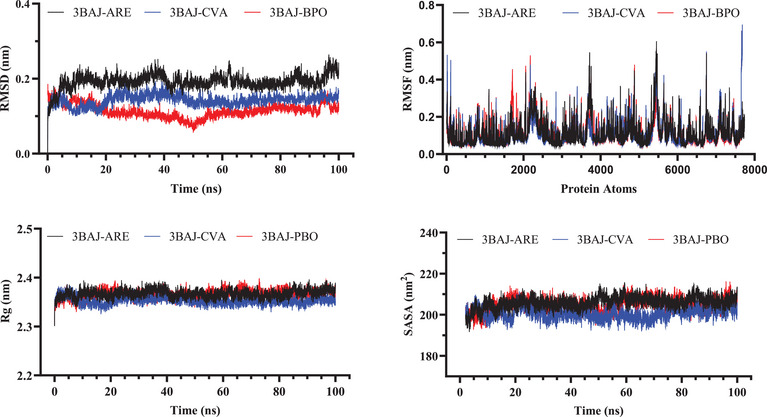
Molecular stability evaluation of α‐amylase complexes.

The Rg analysis of the 3BAJ systems revealed only slight variations in molecular compactness, with values ranging from 2.366 nm (BPO) to 2.367 nm (ARE). The marginally lower Rg of CVA (2.33 nm) suggests a slightly more compact structural conformation in comparison to ARE (2.367 nm) and BPO (2.366 nm), indicating subtle differences in the overall flexibility and packing of the complexes. The SASA analysis indicated that the reference complex (205.960 nm^2^) exhibited the highest solvent exposure compared to the BPO (204.735 nm^2^) and CVA (200.651 nm^2^) complexes. This reduction in solvent‐accessible surface area for the ligand‐bound complexes suggests that ligand interaction may contribute to a more compact structural conformation. Figure 7 illustrates the molecular stability evaluation of the pancreatic α‐amylase systems.

Overall, the MD simulations demonstrated that CVA and BPO systems exhibited superior stability within α‐amylase protein compared to the reference drug ARE. This increased stability suggests that these natural compounds establish stronger and more persistent interactions with the enzyme, potentially enhancing their inhibitory effectiveness. Their ability to maintain stable binding throughout the simulation further underscores their potential as promising candidates for α‐amylase inhibition.

In the investigation of the dynamic behavior of α‐glucosidase systems, the average RMSD values for the 3A4A‐BPO and 3A4A‐CVA complexes were originally reported at 0.291 and 0.292 nm, respectively. The reference complex revealed a higher average RMSD of 0.355 nm. This implies that the BPO and CVA complexes had higher structural stability than the reference, since lower RMSD values often reflect less structural variations over time. The RMSF investigation supports this stability by revealing comparable oscillations across all three complexes, with average RMSF values ranging from 0.109 to 0.150 nm. This homogeneity shows constant atomic mobility throughout the complexes, with no significant variances in flexibility, resulting in steady and well‐organized motion.

The Rg study shows that the BPO and CVA complexes were marginally less compact than the reference complex, as shown by their higher Rg values. The reference complex had the lowest Rg (2.432 nm) and seemed more structurally compact. This indicates that the reference may have greater intramolecular interactions. A lower Rg frequently indicates a more stable and rigid conformation. In contrast, the somewhat higher Rg values of the BPO and CVA complexes indicate a more relaxed structure. Other structural investigations, however, show that they remain stable.

**FIGURE 8:**
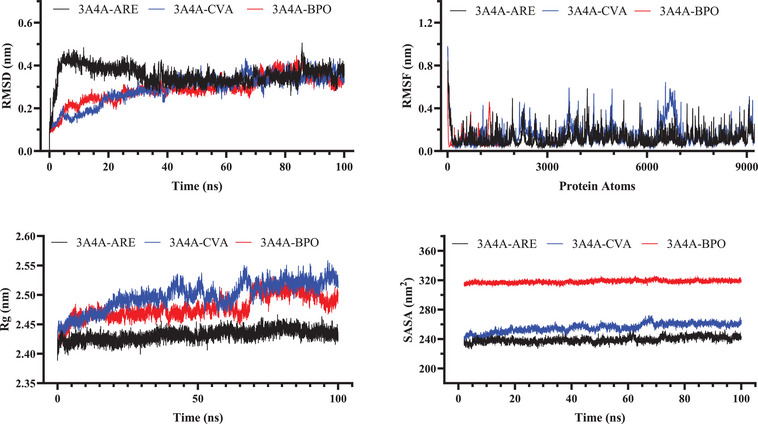
Molecular stability assessment of pancreatic α‐glucosidase systems.

SASA measurements show that the BPO (318.131 nm^2^) and CVA (255.607 nm^2^) complexes were exposed to more solvents than the reference (239.067 nm^2^), which supports the first finding. This implies that BPO and CVA assume more expansive conformations. The higher solvent accessibility might suggest weaker intramolecular interactions. A less compact structural arrangement may contribute to this tendency. In comparison, the reference complex seems more densely packed. This is consistent with its lower Rg value, indicating higher compactness. These findings align with the observed Rg trends. Figure 8 shows the atomic stability evaluation of α‐glucosidase complexes.

MM‐PBSA is a powerful computational method for determining the stability of molecular complexes and calculating binding free energy. It reveals information on the strength and stability of ligand binding by analyzing biomolecular interactions. This technique aids in identifying molecules with high binding affinities. Its capacity to measure interaction energies makes it useful in drug design. MM‐PBSA improves our knowledge of molecular stability in biological systems. Overall, it is a solid approach for estimating binding effectiveness. The total binding energies of CVA, BPO, and ARE in complex with α‐amylase were determined to be –20.12, –19.54, and –18.93 kcal/mol, respectively. Similarly, their binding affinities with α‐glucosidase were calculated as –26.12, –22.54, and –21.93 kcal/mol, respectively. These results indicate strong interactions between the compounds and the enzyme, which may contribute to their inhibitory potential against α‐amylase and α‐glucosidase. Furthermore, the MM‐PBSA calculations align with molecular docking results, reinforcing the strong binding affinities of these natural compounds to α‐amylase and α‐glucosidase. This consistency highlights their potential effectiveness in managing diabetes and mitigating related complications.

#### 3.4.4 DFT Studies

To gain insight into the electronic properties and reactivity of the major compounds in *O. majorana* EO, DFT calculations were performed to determine HOMO, LUMO, and ΔE values in both gas phase and aqueous medium [56]. The results are presented in Table 12, highlighting the influence of solvation on molecular stability and reactivity of the studied molecules.

**TABLE 12:** HOMO, LUMO and ∆E values of the most *O. majorana* EO.

Medium	Parameter	BPO	CVA
Gas	HOMO (eV)	−7.24	−6.64
	LUMO (eV)	−0.29	−0.61
	∆E (eV)	6.95	6.03
Water	HOMO (eV)	−7.41	−6.66
	LUMO (eV)	−0.19	−0.76
	∆E (eV)	7.21	5.90

The HOMO represents the molecule's electron‐donating ability, while the LUMO indicates its tendency to accept electrons. In the gas phase, BPO exhibited a HOMO energy of −7.24 eV, whereas CVA had a higher HOMO energy of −6.64 eV, suggesting that CVA may be more reactive in electron donation compared to BPO. Conversely, the LUMO energy values were −0.29 eV for BPO and −0.61 eV for CVA, indicating that CVA has a slightly greater ability to accept electrons than BPO.

When considering solvent effects, the HOMO energy of BPO decreased slightly to −7.41 eV in water, while its LUMO increased to −0.19 eV. In contrast, CVA exhibited a minor decrease in HOMO to −6.66 eV, while its LUMO energy dropped further to −0.76 eV, indicating a stronger electron‐accepting potential in aqueous conditions.

The ΔE is a critical parameter influencing molecular stability and reactivity. A higher ΔE corresponds to greater molecular stability but lower chemical reactivity, whereas a smaller ΔE suggests higher reactivity and lower stability [57].

In the gas phase, BPO exhibited a ΔE of 6.95 eV, indicating a relatively stable and less reactive nature. CVA had a lower ΔE of 6.03 eV, implying higher chemical reactivity and a greater potential for interactions in biological and chemical environments. Upon solvation, the energy gap of BPO increased to 7.21 eV, suggesting enhanced stability in aqueous conditions. Conversely, CVA's ΔE decreased to 5.90 eV, reinforcing its higher reactivity in water.

The results indicate that CVA exhibits greater reactivity than BPO, particularly in aqueous environments, which may enhance its biological activity and interaction potential. This aligns with molecular docking and molecular dynamics simulations, where CVA demonstrated a higher docking score and greater stability compared to BPO. The variations in HOMO, LUMO, and ΔE values further underscore the distinct electronic properties of these compounds, which are likely to influence their chemical behavior and bioactivity.

The distribution of the HOMO and LUMO plays a crucial role in determining the electron‐donating and electron‐accepting regions, which are key for molecular recognition and binding interactions.

**FIGURE 9:**
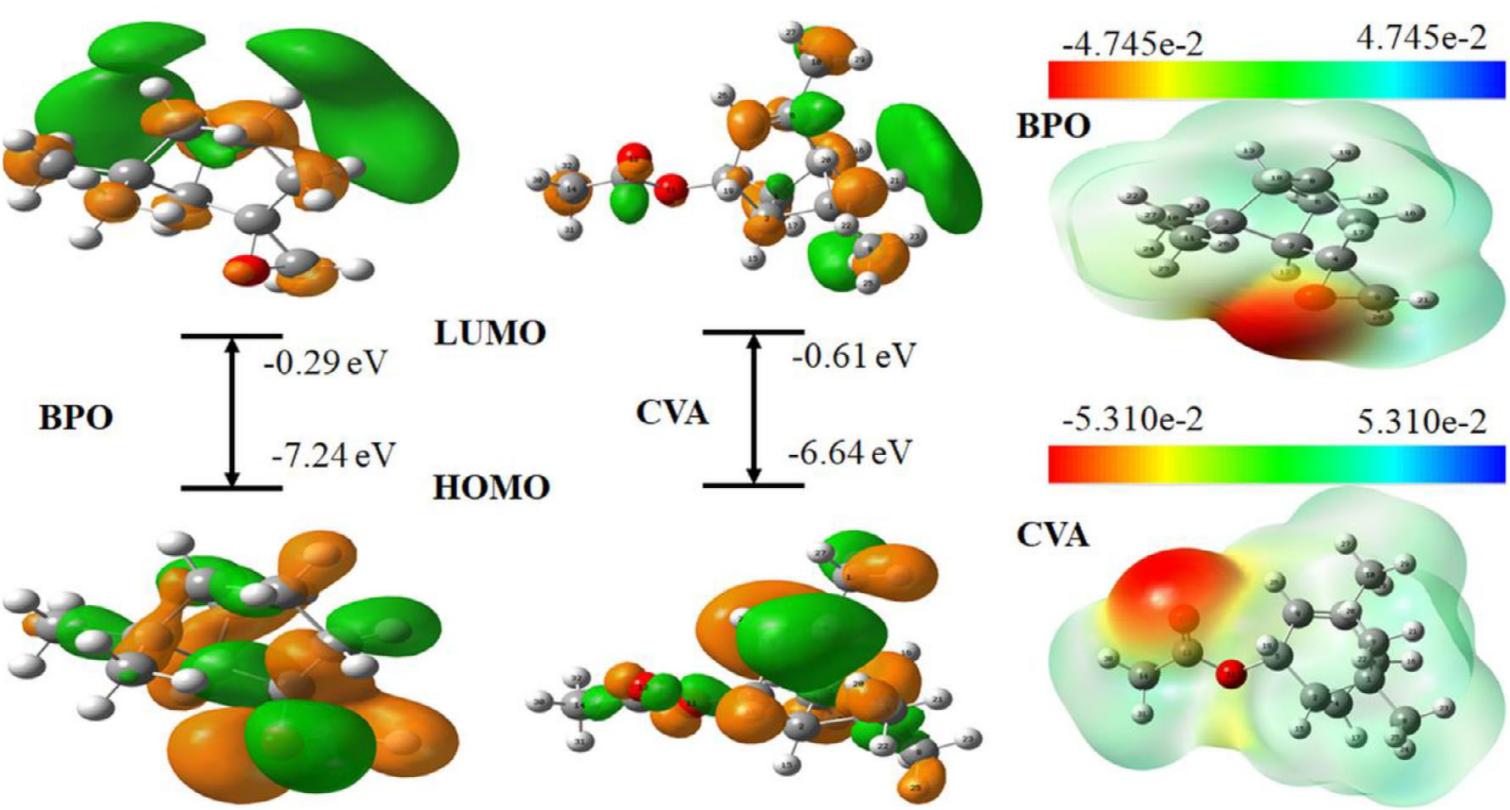
FMO and MEP surface of CVA and BPO in gas phase.

In the gas phase, the HOMO of BPO is distributed across the entire molecule, while the LUMO is primarily localized on the bicycloheptane group (Figure 9). However, in the aqueous phase, the HOMO remains concentrated on the bicycloheptane and oxirane moieties, though slight alterations in its shape suggest solvent‐induced stabilization. The LUMO, in contrast, undergoes a more pronounced redistribution, becoming delocalized across the entire molecule except for the oxirane group (Figure S3).

In the gas phase, the HOMO of BPO is distributed across the entire molecule, while the LUMO is primarily localized on the bicycloheptane group (Figure 9). However, in the aqueous phase, the HOMO remains concentrated on the bicycloheptane and oxirane moieties, though slight alterations in its shape suggest solvent‐induced stabilization. The LUMO, in contrast, undergoes a more pronounced redistribution, becoming delocalized across the entire molecule except for the oxirane group (Figure S3).

For CVA, the HOMO remains delocalized over the entire molecule in both gas and aqueous phases (Figure 9). However, while the LUMO in the gas phase is distributed across the whole molecule, in water, it shifts towards more polar regions, particularly on the acetate and bicycloheptane groups (Figure S3). This redistribution reinforces solute‐solvent interactions, indicating enhanced stabilization in the aqueous environment.

This MEP surface analysis provides valuable insights into the reactive sites of CVA and BPO, reinforcing their potential roles in enzyme inhibition. The MEP map visually represents charge distribution, highlighting the most electrophilic (red or yellow) and nucleophilic (blue) regions within each molecule.

For BPO, in both gas and aqueous phases, the most negative electrostatic potential is concentrated around the oxygen of the oxirane group (Figures 9 and S3). This region, being highly electron‐rich, is likely to participate in interactions with electrophilic species, including active‐site residues of target enzymes. In contrast, CVA exhibits its most negative electrostatic potential around the carbonyl (C = O) functional group (Figures 9 and S3), indicating a distinct reactivity pattern compared to BPO.

The positive electrostatic potential regions in both molecules are relatively well‐distributed, except around the oxygen atoms. This even distribution suggests that both compounds can engage in multiple types of interactions, including hydrophobic and van der Waals forces, which contribute to the stability of the enzyme‐ligand complex.

The presence of water slightly modulates these electrostatic properties but does not significantly alter the fundamental reactive nature of these compounds. These findings align with the FMO analysis, further supporting BPO and CVA as strong candidates for α‐amylase and α‐glucosidase inhibition. Their electronic properties, in conjunction with favorable MEP distributions, highlight their ability to effectively bind to these key metabolic enzymes, making them promising candidates for further exploration in diabetes management.

## 4 Conclusion

This study comprehensively explored the chemical composition and anti‐diabetic potential of *O. majorana* essential oil (EO) through a combination of experimental and computational approaches. GC‐MS analysis identified 42 major components. The anti‐diabetic activity of the EO was evaluated through both in vitro and in vivo assays, demonstrating its ability to inhibit key carbohydrate‐hydrolyzing enzymes, α‐amylase and α‐glucosidase, thereby supporting its potential role in glucose metabolism regulation.

Among the major compounds, molecular docking studies confirmed strong binding affinities with these enzymes. Notably, five compounds exhibited binding scores comparable to the reference drug acarbose (ARE). Complementary ADMET analysis further reinforced these findings, with cis‐verbenyl acetate (CVA) and β‐pinene oxide (BPO) displaying favorable pharmacokinetic properties, drug‐likeness, and low toxicity risks, underscoring the EO's potential for safe and effective therapeutic applications.

Molecular dynamics (MD) simulations validated these results, demonstrating that CVA and BPO formed highly stable complexes with both enzymes, exhibiting greater structural stability than ARE, as evidenced by lower RMSD and RMSF values. MM‐PBSA calculations further confirmed their strong binding affinities, comparable to those of ARE in both enzyme systems.

DFT calculations provided deeper insights into the electronic properties of CVA and BPO, shedding light on their chemical reactivity and interaction potential. The calculated energy gap values indicated moderate reactivity for both compounds, with a slight reduction in the energy gap in water, suggesting enhanced stability and a higher potential for biological interactions under physiological conditions. These findings aligned with molecular docking and MD simulations, which demonstrated that CVA had a more favorable docking score and enhanced stability within the active sites of α‐amylase and α‐glucosidase. Additionally, MEP analysis highlighted key reactive regions, reinforcing their potential for enzyme inhibition.

The combined in vitro, in vivo, and computational findings strongly suggest that *O. majorana* EO, particularly its bioactive constituents CVA and BPO, holds promise as a natural anti‐diabetic agent.

## Author Contributions

**Mahmoud Houas**: conceptualization, data curation, formal analysis, writing – original draft. **Ouafa Boudebia**: visualization. **Aicha Adaika**: writing – review and editing. **Elhafnaoui Lanez**: data curation, formal analysis. **Mohammed Larbi Benamor**: methodology, formal analysis, validation. **Nafila Zouaghi**: resources, supervision. **Lazhar Bechki**: formal analysis. **Yahia Bekkar**: methodology, formal analysis. **Salah Neghmouche Nacer**: writing – review and editing, supervision. **Lotfi Bourougaa**: data curation. **Rania Bouraoui**: formal analysis, visualization. **Touhami Lanez**: funding acquisition, project administration, supervision. **Stefania Garzoli**: writing – review and editing, supervision.

## Funding

This research did not receive any specific grant from funding agencies in the public, commercial, or not‐for‐profit sectors.

## Conflicts of Interest

The authors declare no conflicts of interest.

## Supporting information




**Supporting File**: cbdv71277‐sup‐0001‐SuppMat.docx

## Data Availability

The data will be available upon request to the authors.

## References

[1] J. Reed , S. Bain , and V. Kanamarlapudi , “A Review of Current Trends With Type 2 Diabetes Epidemiology, Aetiology, Pathogenesis, Treatments and Future Perspectives,” Diabetes, Metabolic Syndrome and Obesity 14 (2021): 3567–3602, 10.2147/DMSO.S319895. PMC8369920 34413662

[2] K. Aouadi , H. Hajlaoui , S. Arraouadi , S. Ghannay , M. Snoussi , and A. Kadri , “Phytochemical Profiling, Antimicrobial and α‐Glucosidase Inhibitory Potential of Phenolic‐Enriched Extracts of the Aerial Parts From Echium *humile desf*.: In Vitro Combined With In Silico Approach,” Plants 11 (2022): 1131, 10.3390/plants11091131. 35567133 PMC9105953

[3] T. Hiyoshi , M. Fujiwara , and Z. Yao , “Postprandial Hyperglycemia and Postprandial Hypertriglyceridemia in Type 2 Diabetes,” The Journal of Biomedical Research 33 (2019): 1–16, 10.7555/JBR.31.20160164. PMC6352876 29089472

[4] C. Peyrot des Gachons and P. A. S. Breslin , “Salivary Amylase: Digestion and Metabolic Syndrome,” Current Diabetes Reports 16 (2016): 102, 10.1007/s11892-016-0794-7. 27640169 PMC6825871

[5] M. I. Kazeem , J. O. Adamson , and I. A. Ogunwande , “Modes of Inhibition of α ‐Amylase and α ‐Glucosidase by Aqueous Extract of *Morinda lucida* Benth Leaf,” BioMed Research International 2013 (2013): 1–6, 10.1155/2013/527570. PMC3884628 24455701

[6] S. A. Stein , E. M. Lamos , and S. N. Davis , “A Review of the Efficacy and Safety of Oral Antidiabetic Drugs,” Expert Opinion on Drug Safety 12 (2013): 153–175, 10.1517/14740338.2013.752813. 23241069 PMC3977601

[7] N. Van Quan , H.‐D. Tran , T. D. Xuan , et al., “Momilactones A and B Are α‐Amylase and α‐Glucosidase Inhibitors,” Molecules 24 (2019): 482, 10.3390/molecules24030482. 30700006 PMC6385104

[8] R. Tundis , M. R. Loizzo , and F. Menichini , “Natural Products as α‐Amylase and α‐Glucosidase Inhibitors and Their Hypoglycaemic Potential in the Treatment of Diabetes: An Update,” Mini‐Reviews in Medicinal Chemistry 10 (2010): 315–331, 10.2174/138955710791331007. 20470247

[9] S. Ahmed , C. Ali , R. A. Ruma , et al., “Molecular Docking and Dynamics Simulation of Natural Compounds From Betel Leaves (Piper betle L .) for Investigating the Potential Inhibition of Alpha‐Amylase and Alpha‐Glucosidase of Type 2 Diabetes,” Molecules 27 (2022): 4526. 35889399 10.3390/molecules27144526 PMC9316265

[10] E. L. Chávez‐Delgado and D. A. Jacobo‐Velázquez , “Essential Oils: Recent Advances on Their Dual Role as Food Preservatives and Nutraceuticals Against the Metabolic Syndrome,” Foods 12 (2023): 1079, 10.3390/foods12051079. 36900596 PMC10000519

[11] H. Oberacher , G. Whitley , and B. Berger , “Evaluation of the Sensitivity of the ‘Wiley Registry of Tandem Mass Spectral Data, MSforID’With MS/MS Data of the 'NIST/NIH/EPA Mass Spectral Library,” Journal of Mass Spectrometry 48 (2013): 487–496. 23584942 10.1002/jms.3184

[12] E. Deveci , G. T. E. L. Cayan , and M. E. Duru , “In Vitro Antidiabetic Activity of Seven Medicinal Plants Naturally Growing in Turkey,” European Journal of Biology 79 (2020): 23–28, 10.26650/EurJBiol.2020.0011.

[13] J.‐S. Kim , C.‐S. Kwon , and K. H. Son , “Inhibition of Alpha‐Glucosidase and Amylase by Luteolin, a Flavonoid,” Bioscience, Biotechnology, and Biochemistry 64 (2000): 2458–2461, 10.1271/bbb.64.2458. 11193416

[14] G. M. Morris , R. Huey , W. Lindstrom , et al., “AutoDock4 and AutoDockTools4: Automated Docking With Selective Receptor Flexibility,” Journal of Computational Chemistry 30 (2009): 2785–2791, 10.1002/jcc.21256. 19399780 PMC2760638

[15] H. M. Berman , “The Protein Data Bank,” Nucleic Acids Research 28 (2000): 235–242, 10.1093/nar/28.1.235. 10592235 PMC102472

[16] N. M. O'Boyle , M. Banck , C. A. James , C. Morley , T. Vandermeersch , and G. R. Hutchison , “Open Babel: An Open Chemical Toolbox,” Journal of Cheminformatics 3 (2011): 33, 10.1186/1758-2946-3-33. 21982300 PMC3198950

[17] S. Kim , J. Chen , T. Cheng , et al., “PubChem 2023 Update,” Nucleic Acids Research 51 (2023): D1373–D1380, 10.1093/nar/gkac956. 36305812 PMC9825602

[18] R. Bendaas , L. Messaadia , Y. Bekkar , L. Bourougaa , and A. Messaoudi , “Comprehensive In Vitro and Molecular Docking Analysis of Antioxidant and Antimicrobial Properties of Salvia officinalis L. extracts and Essential Oils,” Journal of Molecular Structure 1331 (2025): 141621, 10.1016/j.molstruc.2025.141621.

[19] M. L. Ben Amor , E. Lanez , Y. Bekkar , et al., “Exploring the Interactions of Ferrocenylmethylnucleobase Derivatives With BSA and HHb: Insights From Electrochemical, Spectroscopic, ADMET, In Silico Docking, and MD Simulations,” ChemistrySelect 10 (2025): 1–16, 10.1002/slct.202404678.

[20] A. Adaika , Y. Bekkar , S. Youmbai , et al., “Synthesis, Antioxidant, and Antidiabetic Potential of Ferrocenylmethylnucleobase Compounds: In Vitro, In Silico Molecular Docking, DFT Calculation, and Molecular Dynamic Simulations,” Applied Organometallic Chemistry 39 (2025): e7988, 10.1002/aoc.7988.

[21] R. Bendaas , Y. Bekkar , L. Messaadia , et al., “Computational‐Based Investigation of Antioxidative Potential Polyphenolic Compounds of *Salvia officinalis L*.: Combined DFT and Molecular Docking Approaches,” Journal of Molecular Modeling 30 (2024): 87, 10.1007/s00894-024-05866-8. 38416254

[22] BIOVIA, *Dassault Systèmes*, Discovery Studio Visualizer, v21.1.0.20298; San Diego: Dassault Systèmes, (2020). https://discover.3ds.com/discovery‐studio‐visualizer‐download (accessed February 18, 2025).

[23] Z. Lalmi , L. Bechki , E. Lanez , and T. Lanez , “Synthesis, Characterization, Cyclic Voltammetry, and Molecular Docking Studies of the Antioxidant Activities of Superoxide Anion Radicals Towards Meso‐Tetramethophenyl‐Porphyrin and Meso‐Tetrabiphenyl‐Porphyrin,” Notulae Scientia Biologicae 16 (2024): 11823, 10.55779/nsb16211823.

[24] V. Zoete , M. A. Cuendet , A. Grosdidier , and O. Michielin , “SwissParam: A Fast Force Field Generation Tool for Small Organic Molecules,” Journal of Computational Chemistry 32 (2011): 2359–2368. 21541964 10.1002/jcc.21816

[25] J. Huang and A. D. MacKerell , “CHARMM36 All‐Atom Additive Protein Force Field: Validation Based on Comparison to NMR Data,” Journal of Computational Chemistry 34 (2013): 2135–2145, 10.1002/jcc.23354. 23832629 PMC3800559

[26] B. Lotfi , O. Mebarka , B. Y. Alhatlani , E. M. Abdallah , and S. M. A. Kawsar , “Pharmacoinformatics and Breed‐Based De Novo Hybridization Studies to Develop New Neuraminidase Inhibitors as Potential Anti‐Influenza Agents,” Molecules 28 (2023): 6678, 10.3390/molecules28186678. 37764457 PMC10534564

[27] N. B. Ayrim , A. A. Balakit , S. J. Laftaa , et al., “Design, Synthesis, and Molecular Docking Studies of 2‐Azetidinone‐Based Combretastatin A‐4 Analogues With Anticancer Activity,” ChemistrySelect 10 (2025): e202405188, 10.1002/slct.202405188.

[28] L. Bourougaa , M. Ouassaf , and S. U. Khan , “Comparative Molecular Field Analysis (CoMFA), Molecular Docking and ADMET Study on Thiazolidine‐4‐carboxylic Acid Derivatives as New Neuraminidase Inhibitors,” Acta Chimica Slovenica 70 (2023): 333–344, 10.17344/acsi.2023.8111. 40836548

[29] L. Bourougaa , M. Ouassaf , and B. Y. Alhatlani , “Clinical Informatics and Molecular Hybridization of Established Clinical DPP‐4 Inhibitors to Generate Next‐Level Diabetes Type 2 Drugs,” Chemical Papers 78 (2024): 8485–8503, 10.1007/s11696-024-03697-8.

[30] L. Bourougaa , M. Ouassaf , and A. Shtaiwi , “Discovery of Novel Potent Drugs for Influenza by Inhibiting the Vital Function of Neuraminidase via Fragment‐based Drug Design (FBDD) and Molecular Dynamics Simulation Strategies,” Journal of Biomolecular Structure & Dynamics 42 (2024): 9294–9308, 10.1080/07391102.2023.2251065. 37640004

[31] A. Mishra , V. Mulpuru , and N. Mishra , “Exploring the Mechanism of Action of Podophyllotoxin Derivatives Through Molecular Docking, Molecular Dynamics Simulation and MM/PBSA Studies,” Journal of Biomolecular Structure and Dynamics 41 (2023): 8856–8865, 10.1080/07391102.2022.2138549. 36307902

[32] R. Kumari , R. Kumar , and A. Lynn , “ *g_mmpbsa* —A GROMACS Tool for High‐Throughput MM‐PBSA Calculations,” Journal of Chemical Information and Modeling 54 (2014): 1951–1962, 10.1021/ci500020m. 24850022

[33] B. Gogoi , P. Chowdhury , N. Goswami , et al., “Identification of Potential Plant‐Based Inhibitor Against Viral Proteases of SARS‐CoV‐2 Through Molecular Docking, MM‐PBSA Binding Energy Calculations and Molecular Dynamics Simulation,” Molecular Diversity 25 (2021): 1963–1977, 10.1007/s11030-021-10211-9. 33856591 PMC8047602

[34] P. H. Riyadi , I. D. S. Romadhon , R. A. Kurniasih , et al., “SwissADME Predictions of Pharmacokinetics and Drug‐Likeness Properties of Small Molecules Present in Spirulina Platensis,” IOP Conference Series: Earth and Environmental Science 890 (2021): 2063–2073, 10.1088/1755-1315/890/1/012021.

[35] P. Banerjee , A. O. Eckert , A. K. Schrey , and R. Preissner , “ProTox‐II: A Webserver for the Prediction of Toxicity of Chemicals,” Nucleic Acids Research 46 (2018): W257–W263, 10.1093/nar/gky318. 29718510 PMC6031011

[36] E. U. A.‐R.‐S. R. P. Frisch, T. M.J., S. G.W., et al., *Gaussian* 16 (2016), Revision B.01, (, ) Gaussian 16, Revision A.03.

[37] A. D. Becke , “Density‐Functional Thermochemistry. III. The Role of Exact Exchange,” The Journal of Chemical Physics 98 (1993): 5648–5652, 10.1063/1.464913.

[38] L. Messaadia , Y. Bekkar , M. Benamira , and H. Lahmar , “Predicting the Antioxidant Activity of Some Flavonoids of Arbutus Plant: A Theoretical Approach,” Chemical Physics Impact 1 (2020): 100007, 10.1016/j.chphi.2020.100007.

[39] R. Dennington , T. A. Keith , and J. M. Millam , *GaussView, Version* 6 (2016).

[40] M. L. Swift , “GraphPad Prism, Data Analysis, and Scientific Graphing,” Journal of Chemical Information and Computer Sciences 37 (1997): 411–412, 10.1021/ci960402j.

[41] O. Boudebia , M. L. Benamor , L. Bourougaa , et al., “Holistic Investigation of Cotula cinerea Essential Oil Against Diabetes: Hypoglycemic Activity, Enzymatic Inhibition, GC‐MS Characterization, ADMET Forecasting, MD Simulations, and DFT Insights,” Journal of Computer‐Aided Molecular Design 39 (2025): 80, 10.1007/s10822-025-00664-7. 40952536

[42] E. Lanez , Y. Bekkar , L. Bourougaa , et al., “Antidiabetic Potential of Mentha Piperita Essential Oil: GC‐MS Profiling, In Vitro, In Vivo and In Silico Analyses,” Journal of Molecular Structure 1351 (2025): 144239.

[43] O. M. Ighodaro , A. M. Adeosun , and O. A. Akinloye , “Alloxan‐Induced Diabetes, a Common Model for Evaluating the Glycemic‐Control Potential of Therapeutic Compounds and Plants Extracts in Experimental Studies,” Medicina (B Aires) 53 (2017): 365–374, 10.1016/j.medici.2018.02.001. 29548636

[44] S. Yamada and H. Asakura , “How We Interpret Thrombosis With Thrombocytopenia Syndrome?,” International Journal of Molecular Sciences 25 (2024): 4956, 10.3390/ijms25094956. 38732176 PMC11084439

[45] F. A. Matough , S. B. Budin , Z. A. Hamid , N. Alwahaibi , and J. Mohamed , “The Role of Oxidative Stress and Antioxidants in Diabetic Complications,” Sultan Qaboos University Medical Journal 12 (2012): 5–18, 10.12816/0003082. 22375253 PMC3286717

[46] L. Dilworth , A. Facey , and F. Omoruyi , “Diabetes Mellitus and Its Metabolic Complications: The Role of Adipose Tissues,” International Journal of Molecular Sciences 22 (2021): 7644, 10.3390/ijms22147644. 34299261 PMC8305176

[47] A. Dhillon and R. H. Steadman , “Liver Diseases,” in Anesthesia and Uncommon Diseases (Elsevier, 2012): 162–214, 10.1016/B978-1-4377-2787-6.00005-X.

[48] N. A. A. Amartey , “Plasma Levels of Uric Acid, Urea and Creatinine in Diabetics Who Visit the Clinical Analysis Laboratory (CAn‐Lab) at Kwame Nkrumah University of Science and Technology, Kumasi, Ghana,” Journal of Clinical and Diagnostic Research 9 (2015): BC05, 10.7860/JCDR/2015/10905.5530. PMC4378725 25859443

[49] H. A. Benesi and J. H. Hildebrand , “A Spectrophotometric Investigation of the Interaction of Iodine With Aromatic Hydrocarbons,” Journal of the American Chemical Society 71 (1949): 2703–2707, 10.1021/ja01176a030.

[50] S. Alqahtani , “In Silico ADME‐Tox Modeling: Progress and Prospects,” *Expert Opinion on Drug Metabolism & Toxicology* 13 (2017): 1147–1158, 10.1080/17425255.2017.1389897. 28988506

[51] E. Lanez , T. Lanez , and N. Zegheb , “In Silico ADMET, Toxicological Analysis, Molecular Docking Studies and Molecular Dynamics Simulation of Afzelin With Potential Antibacterial Effects Against *Staphylococcus aureus* ,” Turkish Computational and Theoretical Chemistry 7 (2023): 10–16, 10.33435/tcandtc.1196422.

[52] C. A. Lipinski , F. Lombardo , B. W. Dominy , and P. J. Feeney , “Experimental and Computational Approaches to Estimate Solubility and Permeability in Drug Discovery and Development Settings,” Advanced Drug Delivery Reviews 23 (1997): 3–25, 10.1016/S0169-409X(96)00423-1. 11259830

[53] S. Khamouli , S. Belaidi , M. Ouassaf , T. Lanez , S. Belaaouad , and S. Chtita , “Multi‐Combined 3D‐QSAR, Docking Molecular and ADMET Prediction of 5‐Azaindazole Derivatives as LRRK2 Tyrosine Kinase Inhibitors,” Journal of Biomolecular Structure and Dynamics 40 (2022): 1285–1298, 10.1080/07391102.2020.1824815. 32964807

[54] Z. Bibi , “Role of Cytochrome P450 in Drug Interactions,” Nutrition & Metabolism 5 (2008): 27, 10.1186/1743-7075-5-27. 18928560 PMC2584094

[55] E. Lanez , A. Kedadra , T. Lanez , A. Adaika , and N. Zegheb , “In Vitro Antioxidant Activity, QSAR, In Silico Toxicity Prediction, Molecular Docking Studies, and Molecular Dynamics of a Series of N‐ferrocenylmethylanilines as Potent Antioxidant Agents,” Journal of Organometallic Chemistry 1017 (2024): 123284, 10.1016/j.jorganchem.2024.123284.

[56] K. Fukui , “Role of Frontier Orbitals in Chemical Reactions,” Science 218 (1979): 747–754, 10.1126/science.218.4574.747. 17771019

[57] M. M. Hammoud , M. Khattab , M. Abdel‐Motaal , et al., “Synthesis, Structural Characterization, DFT Calculations, Molecular Docking, and Molecular Dynamics Simulations of a Novel Ferrocene Derivative to Unravel Its Potential Antitumor Activity,” Journal of Biomolecular Structure and Dynamics 11 (2023): 5199–5216, 10.1080/07391102.2022.2082533. 35674744

