# Supplementary material for: Therapeutic Potential of Origanum majorana L. Essential Oil in Diabetes Mellitus: Insights From GC–MS Characterization, In Vivo Hypoglycaemic Studies, and In Silico Analyses
Source: Chem Biodivers. 2026 May 4;23:e71277. doi: 10.1002/cbdv.71277 (PMC13138695; doi:10.1002/cbdv.71277)
Supplement: Supplementary file 1 — Supporting File: cbdv71277‐sup‐0001‐SuppMat.docx [file CBDV-23-e71277-s001.docx]

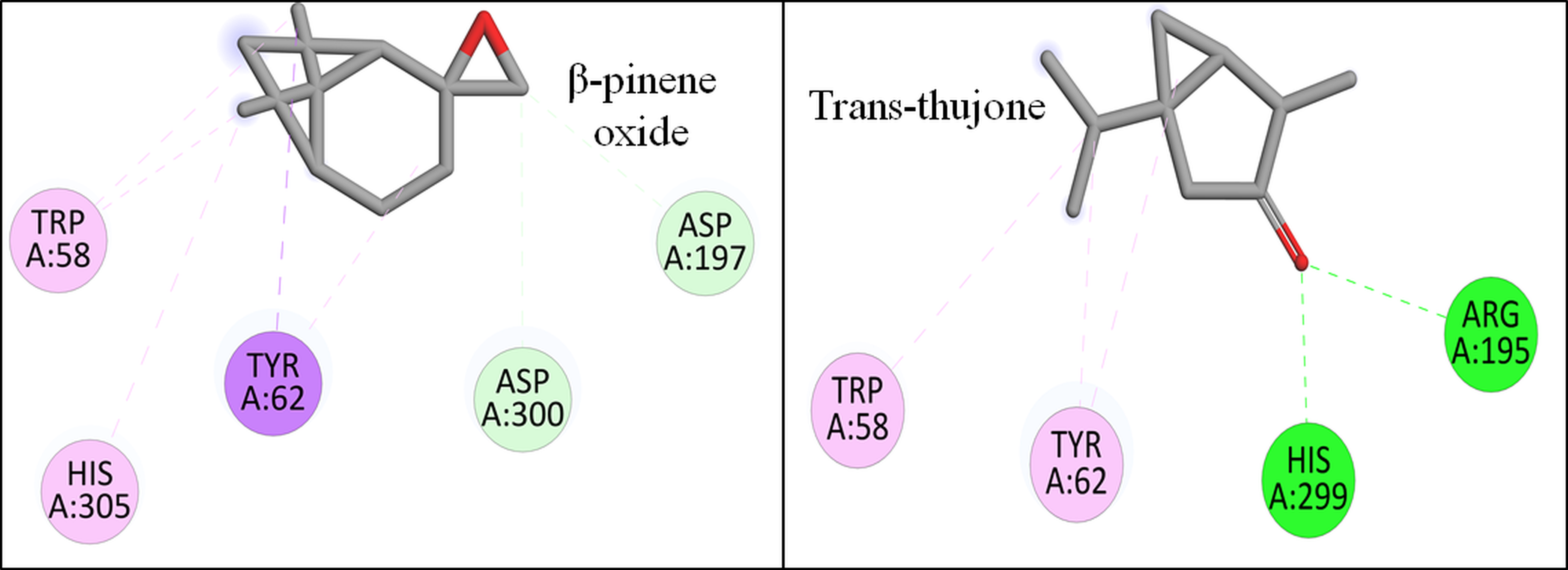


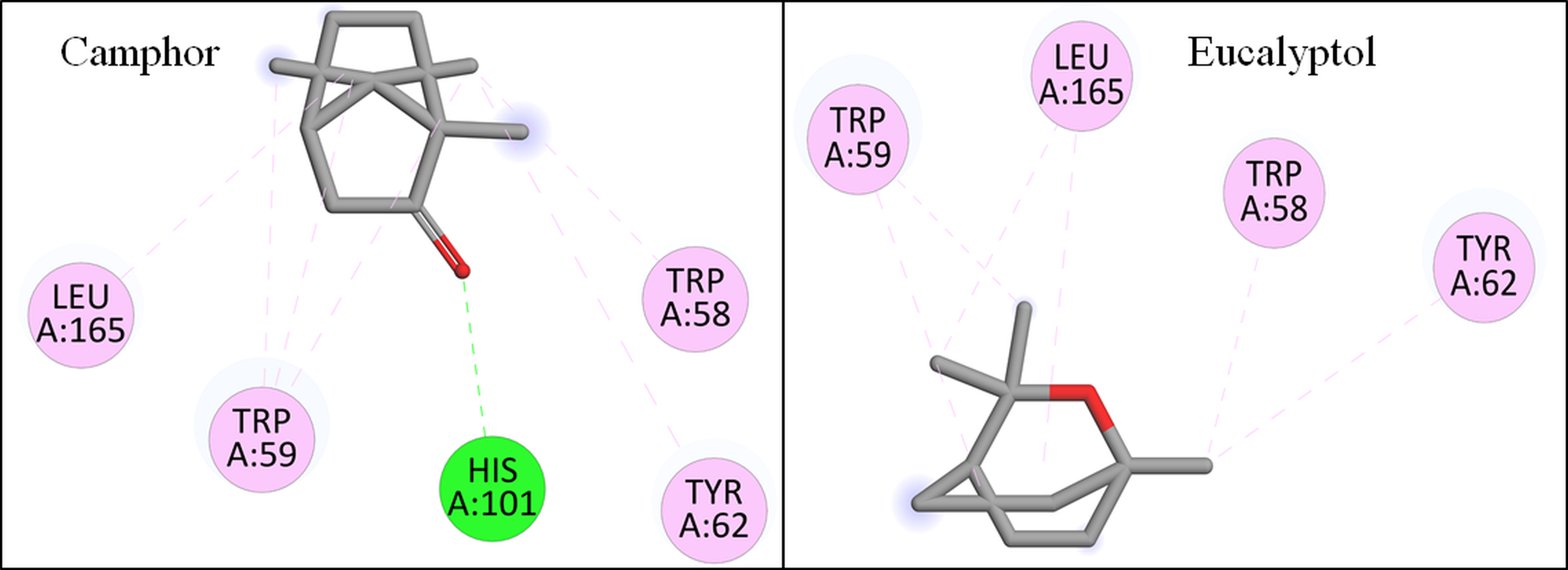


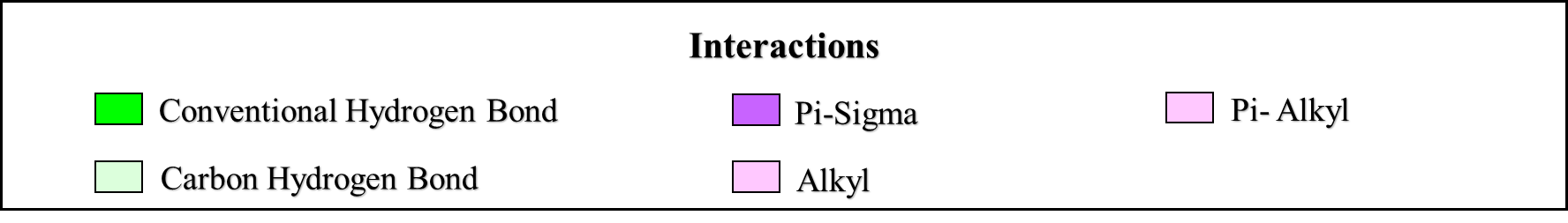


**Figure S1.** 2D Binding interactions of selected *O. majorana* EO compounds with the active site of α-amylase


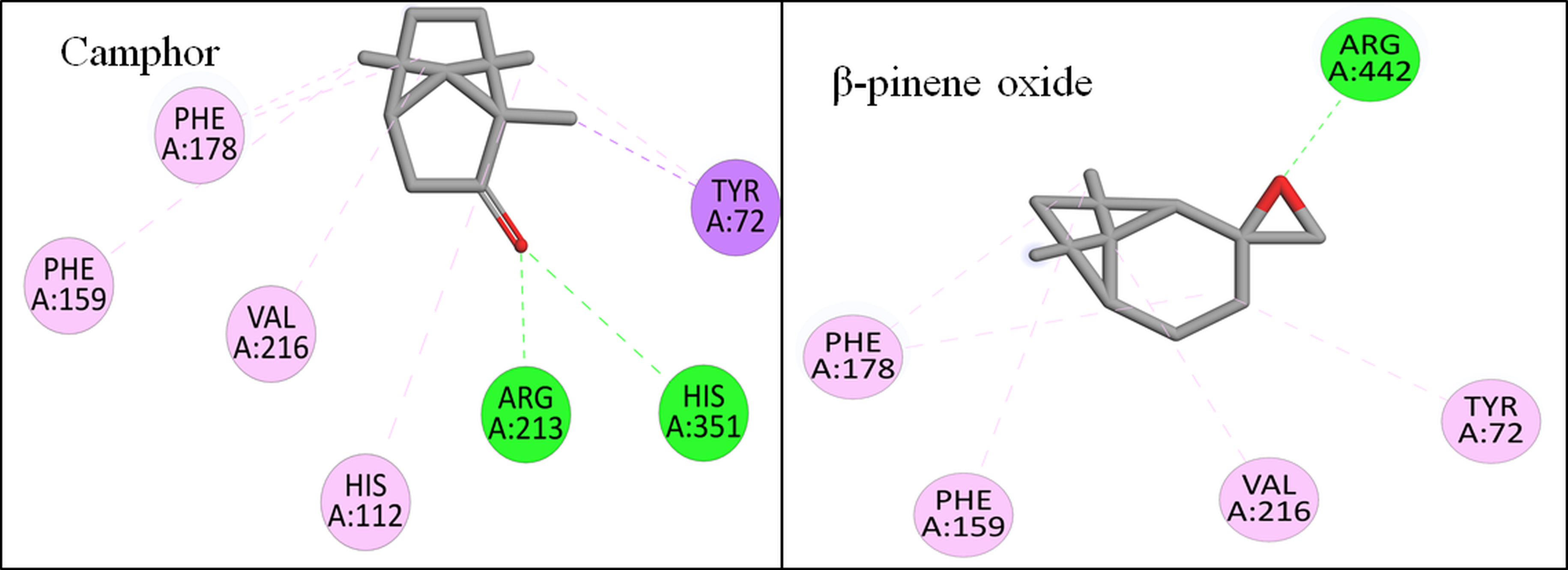


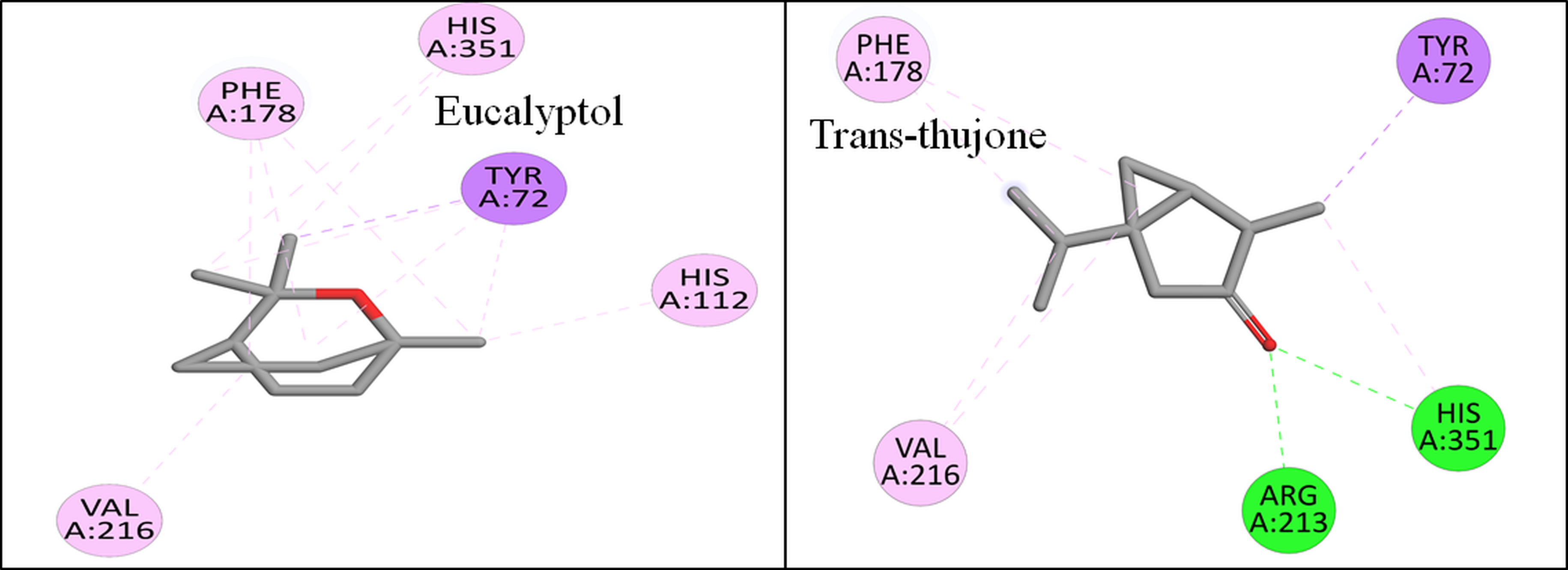


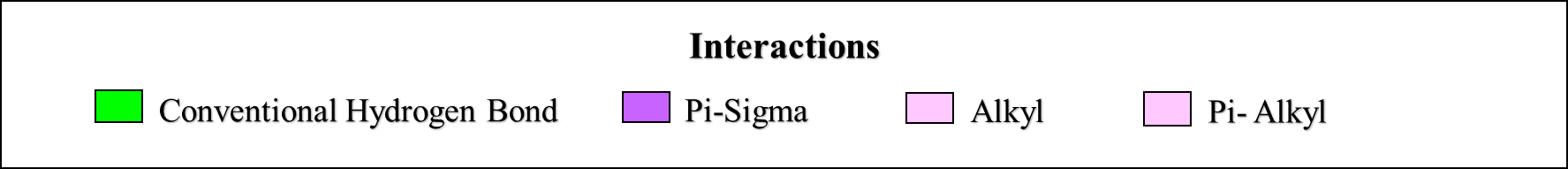


**Figure S2**. 2D Binding interactions of selected *O. majorana* EO compounds with the active site of α-glucosidase


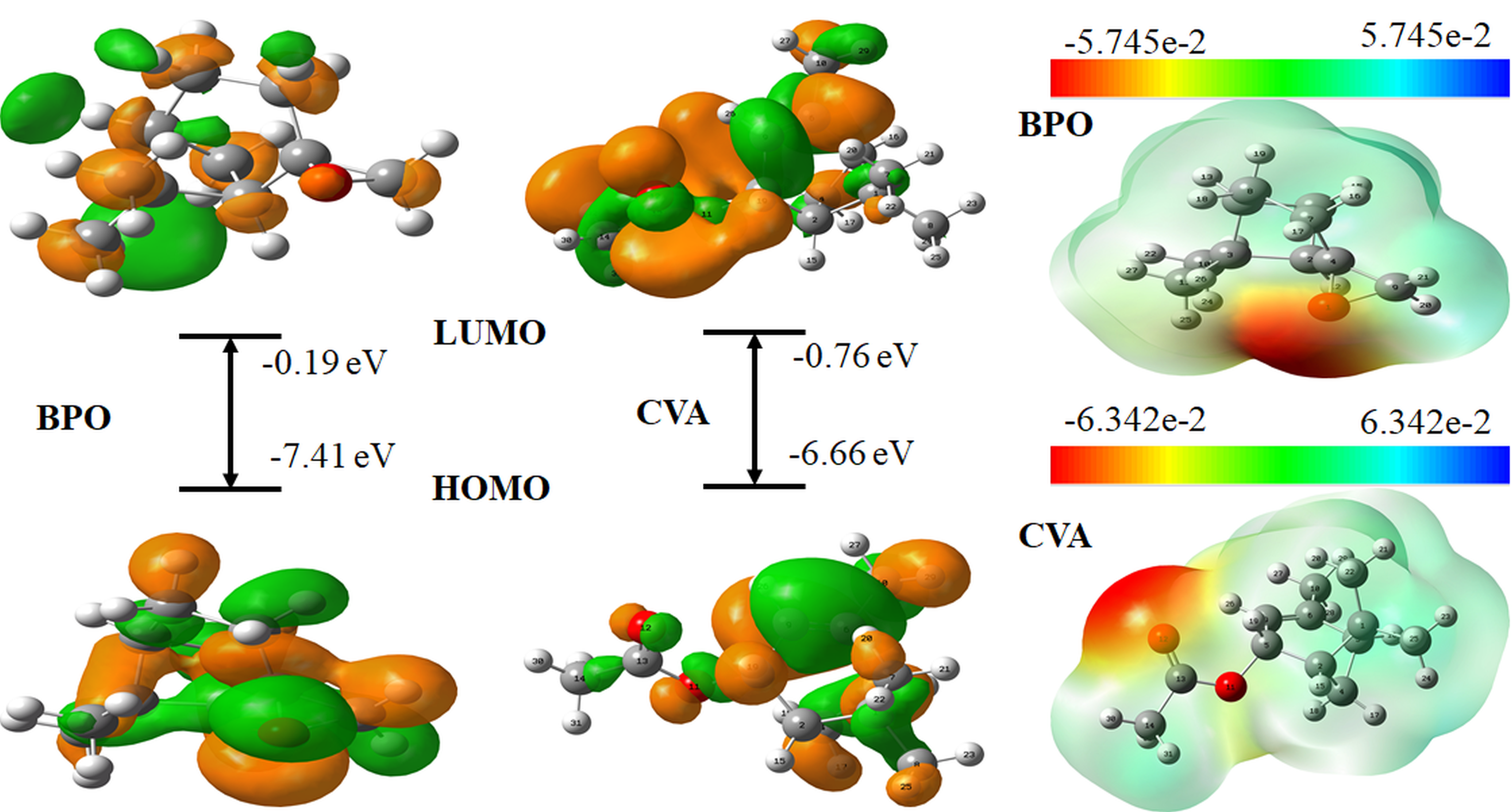


**Figure S3**. FMO and MEP surface of CVA and BPO in water phase
